# Multi-time series RNA-seq analysis of *Enterobacter lignolyticus* SCF1 during growth in lignin-amended medium

**DOI:** 10.1371/journal.pone.0186440

**Published:** 2017-10-19

**Authors:** Roberto Orellana, Gina Chaput, Lye Meng Markillie, Hugh Mitchell, Matt Gaffrey, Galya Orr, Kristen M. DeAngelis

**Affiliations:** 1 Centro de Biotecnología Daniel Alkalay Lowitt, Universidad Técnica Federico Santa María, Valparaíso, Chile; 2 Microbiology Department, University of Massachusetts Amherst, Amherst, United States of America; 3 Pacific Northwest National Laboratory, Richland, Washington, United States of America; Hubei University, CHINA

## Abstract

The production of lignocellulosic-derived biofuels is a highly promising source of alternative energy, but it has been constrained by the lack of a microbial platform capable to efficiently degrade this recalcitrant material and cope with by-products that can be toxic to cells. Species that naturally grow in environments where carbon is mainly available as lignin are promising for finding new ways of removing the lignin that protects cellulose for improved conversion of lignin to fuel precursors. *Enterobacter lignolyticus* SCF1 is a facultative anaerobic Gammaproteobacteria isolated from tropical rain forest soil collected in El Yunque forest, Puerto Rico under anoxic growth conditions with lignin as sole carbon source. Whole transcriptome analysis of SCF1 during *E*.*lignolyticus* SCF1 lignin degradation was conducted on cells grown in the presence (0.1%, w/w) and the absence of lignin, where samples were taken at three different times during growth, beginning of exponential phase, mid-exponential phase and beginning of stationary phase. Lignin-amended cultures achieved twice the cell biomass as unamended cultures over three days, and in this time degraded 60% of lignin. Transcripts in early exponential phase reflected this accelerated growth. A complement of laccases, aryl-alcohol dehydrogenases, and peroxidases were most up-regulated in lignin amended conditions in mid-exponential and early stationary phases compared to unamended growth. The association of hydrogen production by way of the formate hydrogenlyase complex with lignin degradation suggests a possible value added to lignin degradation in the future.

## Introduction

One of the key challenges that environmental sciences currently face is the search for renewable energy sources that are capable to satisfy future energy demands. Among several choices, biofuels derived from biomass have emerged as one of the most promising for decreasing the harmful effects produced by the uncontrolled use of fossil fuels [1, 2]. Lignocellulosic biomass represents close to the 90% of the dry weight of the total biomass material [3, 4]. Lignocellulose is composed of cellulosic and hemicellulosic polysaccharides, pectic polymers, and lignin. Lignin is a phenylpropanoid-derived heteropolymer that comprises 10–30% of the lignocellulosic biomass. The stable and complex physical structure of lignin together with its chemical inhibitory nature confers rigidity to plant tissue and also serving as a protection from pathogens [5, 6]. Due to its aromatic nature and highly branched polymer network, lignin is resistant to biological degradation and it is the primary material responsible for biomass recalcitrance [7]. Efforts aimed to advance lignin bioengineering in combination with novel lignin separation and biodegradation technologies are required to make lignocellulosic biofuels competitive with fossil fuels.

Microbial lignin degradation was thought to be a negligible bioprocess in the environment until 1928 when experiments carried out by Bavendam and collaborators showed that lignin could be biologically depolymerized [8]. These initial studies were supported by several studies carried out with white-rot or brown-rot fungi [9, 10]. In general, those microorganisms utilize extracellular peroxidases and laccases-mediated mechanisms during the aerobic degradation of lignin [5]. More recent studies have shown that lignin monomers such as vanillin, vanillic acid, ferulic acid, catechol and cinnamic acid can be metabolized under both aerobic and anaerobic conditions [11–14]. For instance, the phototrophic *Rhodopseudomonas palustris* [15], and the denitrifying *Thauera aromatica* [16] are well know because of their capability of anaerobic degradation of aromatic compounds. More recent experiments demonstrated that lignin can be degraded in the absence of oxygen [11, 17]. Facultative anaerobes have been shown to be capable of using lignin as the sole carbon source under anaerobic conditions, and we have isolated numerous lignin-degrading bacteria from El Yunque experimental forest, Puerto Rico based on their ability to grow on lignin as sole C source: *Klebsiella* sp. strain BRL6-2 [18], *Tolumonas lignolytica* sp. nov [19], and *Enterobacter lignolyticus* SCF1 [20].

*Enterobacter lignolyticus* SCF1 is a facultative anaerobic Gammaproteobacteria. Its close relative *Enterobacter soli* strain LF7, was also isolated from tropical forest soils in Peru using lignin as sole C source [21]. Although, these soils are characterized by periods of extremely low availability of oxygen and extended periods of low redox [22], tropical forest soils have been shown to have among the fastest rates of plant litter decomposition worldwide [23–25]. *E*. *lignolyticus* SCF1 sparked initial attention due to its ability to grow on lignin as a sole carbon source in the absence of oxygen, as well as its high tolerance to 1-ethyl-3-methylimidazolium chloride, a toxic compound utilized as a lignocellulosic biomass pretreatment in the production of biofuels [26]. These studies employed RNA-seq, a sensitive tool used to better understand the physiological status of bacteria by quantifying genome-wide gene expression [27]. Growth of *E*. *lignolyticus* SCF1 in lignin revealed increased 4-hydroxyphenylacetate degradation pathway, catalase/peroxidase enzymes, and the glutathione biosynthesis and glutathione S-transferase (GST) proteins, which have been previously implicated in anoxic lignin degradation. Growth on lignin was also associated with increased production of NADH-quinone oxidoreductase, other electron transport chain proteins, and ATP synthase and ATP-binding cassette (ABC) transporters, suggesting that lignin-amended conditions include extra reductive capacity in addition to observed oxidative and hydrolytic lignin-reducing enzymes [20].

RNA-seq studies are often conducted as static sampling experiments [28], based on the analysis of transcriptomes of microorganisms facing conditions at a specific time-point during growth. However, microorganisms respond to environmental changes in a dynamic fashion, which was the motivation for examining lignin degradation across three time points corresponding to different stages of growth. Our current experiment also differs from previous experiments due to the different substrate utilized. In former experiments, xylose, a pentose sugar commonly present in hydrolysates from lignocellulosic biomass was utilized as main C source. Those conditions may mimic slow-growing conditions that may be often found in soil environments. However, we observed that SCF-1 grew poorly during those experiments (achieving OD_600_ ~0.03) suggesting that this condition may have generated an additional stress that could obscure the global transcriptional response found when SCF-1 was incubated in the presence of lignin. Indeed, under those conditions, only the 6% (273/4599) of the genes were found to be differentially expressed when lignin was present [20]. In contrast, our current experiments were based on SCF-1 growing in glucose, which was chosen as a fast-growing condition, mimicking environmental conditions encountered in nutrient-rich ecosystems, such as those found by gastrointestinal microbiota. Both conditions differ not only in the affinity of their carbon source, but also in the quantity of energy (ATP) and reducing equivalents that the main pathways are capable to produce (S1 Fig). To improve our understanding of SCF1 lignin degradation over time [20], an experiment was designed to capture the gene expression profile of lignin degradation during growth (early, mid-exponential and beginning of stationary phase of growth) and in the presence and absence of lignin.

## Materials and methods

### Strains and culturing

The facultative anaerobe *E*. *lignolyticus* SCF1 was obtained from our laboratory culture collection. Cells were routinely grown in anaerobic modified LS4D minimal media as previously described [20], with the exception that PIPES (30 mM) was added instead of Tris-Cl (2 mM) as buffer and glucose (60 mM), instead of xylose, was added as sole carbon source. Since these conditions induced higher cell growth of *E*. *lignolyticus* SCF1 than was observed in previous experiments [20], we also duplicated the concentration of lignin supplied at the beginning of growth (0.1% w/w). The lignin source utilized for these experiments was alkali lignin (Sigma 45–471003) that according to the supplier’s specifications, it contained 4% of sulfur impurities. Cell growth was monitored by measuring the optical density at 600 nm (OD_600_) (Genesys 2, Spectronic Instruments, Rochester, NY). Because the lignin-amended media has a spectroscopic signature, lignin-amended cultures are reported with a blank subtraction using lignin-amended media, while unamended cultures are reported with a blank subtraction using unamended media. Lignin concentration was measured by removing 1 ml of culture from anaerobic septum bottles, diluting 1:10 in distilled water, filtering out cells, then measuring the absorbance at 310 nm and compared to a standard curve of known concentrations of lignin, as perfomed before [20, 29].

RNA-seq-based transcriptome analysis was conducted comparing cells grown in glucose (unamended control) to cells grown on both glucose and lignin (lignin-amended treatment). For this analysis, triplicates from the initial nine biological replicates of cells grown in both conditions were destructively sampled anaerobically for transcriptomics analysis at three time points. Samples for the first-time point were collected 3 hours after inoculation in both treatments, in early exponential phase (EE). Mid-exponential phase samples (ME) were collected 7 and 22 hours after inoculation in unamended control and lignin-amended cultures, respectively. The last time point was in early stationary phase (ES), for which samples were taken 22 and 47 hours after inoculation in unamended and lignin-amended cultures.

### Metabolites analysis

High-pressure liquid chromatography (HPLC) to measure glucose, lactate, ethanol, formate, acetate, succinate in culture supernatants were performed on Shimadzu LC-20AD liquid chromatograph with a DGU-20A5 degasser and SIL-20 ACHT autosampler. The HPLC was equipped with an Aminex HPX-87H column, Biorad Microguard Cation H guard column and a RID-10A refractive index detector. The column was kept at 30°C with a flow rate of 0.6mL/min using 5 mM H_2_SO_4_ as the mobile phase. The coefficient of regression (R^2^) of all standards utilized were all over 0.99. All samples were run in triplicate. Lignin measurements were performed as previously described [19, 20]. Briefly, the absorbance of previously filtered (0.22 um) culture supernatants was measured at 310 nm and compared to a standard curve of known concentrations of lignin.

Hydrogen gas production was tested in separate growth conditions compared to cultures for transcriptomic analysis. Resting cell suspensions were prepared by harvesting cells at late exponential phase with an optical density of 0.18–0.2 by centrifugation for 12 minutes at 5000 RPM, and washed twice with washing buffer containing LS4D minimal media with no vitamins, trace elements, and C sources (glucose, lignin) added. Afterwards, cells were added into the washing buffer at a concentration of OD_600_ equal to 0.15, formate (150 mM) and lignin (0.05%) were added as C source and incubated anaerobically at 30°C. Controls were prepared with no addition of C source. Samples (200 μl) of headspace from each bottle at each time point were utilized to measure the amount of hydrogen gas using a gas chromatograph (Shimadzu GC-8A) equipped with a 60/80 Carboxen 1000 column (Supelco), and using Ar as the carrier gas.

### mRNA extraction

Total RNA was extracted from harvested cells using Invitrogen TRIzol^®^ Reagent (cat#15596018), followed by genomic DNA removal and cleaning using Qiagen RNase-Free DNase Set kit (cat#79254) and Qiagen Mini RNeasy^™^ kit (cat#74104). Agilent 2100 Bioanalyzer was used to assess the integrity of the RNA samples. Only RNA samples having RNA Integrity Number between 8–10 were used.

### RNA-sequencing

The Applied Biosystems SOLiD^™^ Total RNA-Seq kit (catalog number 4445374) was used to generate the cDNA template library. The SOLiD^™^ EZ Bead system was used to perform emulsion clonal bead amplification to generate bead templates for SOLiD^™^ platform sequencing. Samples were sequenced on the SOLiD^™^ 4 platform. The 50-base short read sequences produced by the 5500xl SOLiD^™^ sequencer were mapped in color space using SOLiD^™^ LifeScope^™^ software version 2.5 using the default settings to map the short reads onto *Enterobacter lignolyticus* SCF1 (NC_014618.1) reference genome and both the fasta and the GFF files can be obtained from NCBI genome database (http://www.ncbi.nlm.nih.gov/genome). Sequence reads have been submitted to the NCBI databases under accession no GSE96828.

### Statistical analysis

RNA-seq samples from the SOLiD sequencing platform were normalized and processed for differential expression using the edgeR package in Bioconductor [30]. Data was retained only from genes manifesting at least three reads per million in at least three individual replicates. Time points with glucose plus lignin were compared with matched time points with glucose alone. Genes with adjusted p-values <0.05 and absolute value of log2 fold change >1 were deemed differentially expressed for these comparisons. Venn diagrams were constructing using an online tool courtesy of the Bioinformatics and Research Computing group at the Whitehead Institute for Biomedical Research (http://jura.wi.mit.edu/bioc/tools/venn3way/), with the caveat that the sizes of the circles may not precisely be to scale. Histograms were generated with selected genes using the R programming language, with heatmaps made using the gplots package. Hierarchical clustering was performed using hclust function with the “complete” method setting and euclidian distance measures.

## Results and discussion

To gain insight into physiological differences during lignin degradation, the gene transcript abundances in *Enterobacter lignolyticus* SCF1 were compared in cultures grown on glucose minimal media to the same media amended with lignin. Our experimental design was intended to characterize temporal dynamics during the degradation of lignin, as a way of illuminating the series of progressive biological mechanisms involved in lignin degradation. Populations were sampled for transcriptomics analysis at early exponential phase (EE), mid-exponential phase (ME) and early stationary phase (ES).

### Global view of the response to lignin

**Fig 1:**
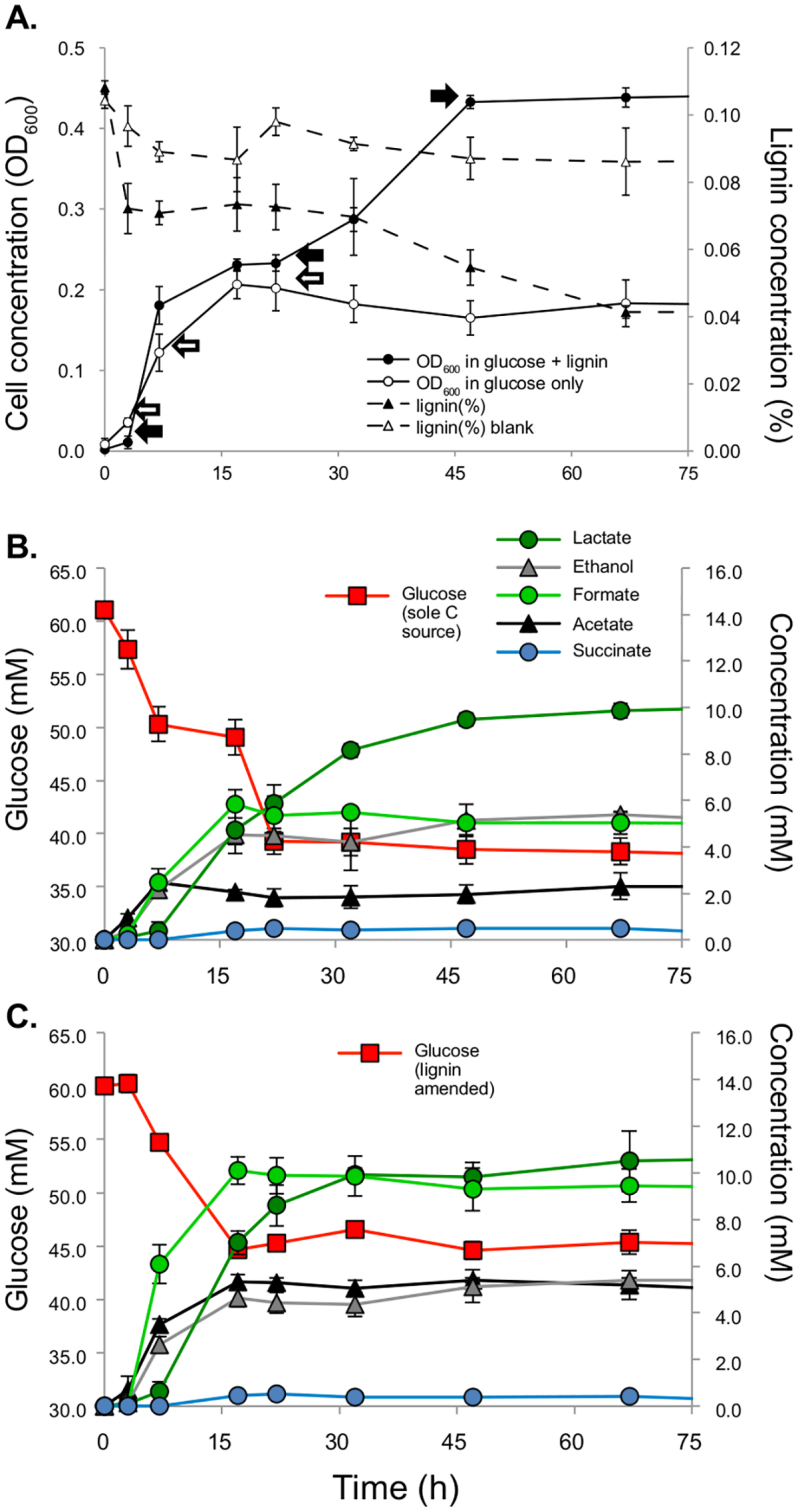
**(A)** Growth curve of *Enterobacter lignolyticus* SCF-1 in glucose medium (open circles) and glucose medium amended with lignin (closed circles). Arrows show the times samples were harvested for transcriptomic analysis (open arrows, glucose medium and closed arrows, glucose medium amended with lignin). The concentration of lignin of amended cultures is shown in closed triangles and control (medium mas lignin, no cells) is shown in open triangles. Metabolites concentrations along growth of unamended cultures **(B)** and lignin amended cultures **(C)**. Glucose is shown in red squares, ethanol, in gray triangles, acetate, in black triangles, lactate, in darker green circles, succinate in light blue and formate, lighter green circles.

SCF-1 achieved twice the cell density when lignin was added in the medium compared to unamended growth (Fig 1A, S2 and S3 Figs). This difference is associated with the degradation of lignin, since in the lignin-amended cultures there was a 40% lower consumption of glucose (~8 mM) than in the unamended cultures (Fig 1B and 1C). It has been previously shown that SCF-1 can utilize lignin as the sole carbon source during anaerobic growth in agar [20, 23]. Although we have been unable to detect growth of SCF-1 as sole C source in liquid medium, it has been previously reported that SCF-1 is capable to degrade lignin up to 56% within 48 hours in the presence of xylose [20]. When growing on lignin-amended LS4D liquid medium in the presence of glucose, there was a first drop in the content of lignin of about 30% (0–30 hours) at the beginning of the growth curve, that resulted in SCF-1 achieving a slightly higher cell concentration than the control. After 30 hours, the concentration of lignin was constantly decreasing until SCF-1 ultimately degraded 60% of the lignin amended in the medium.

**Fig 2:**
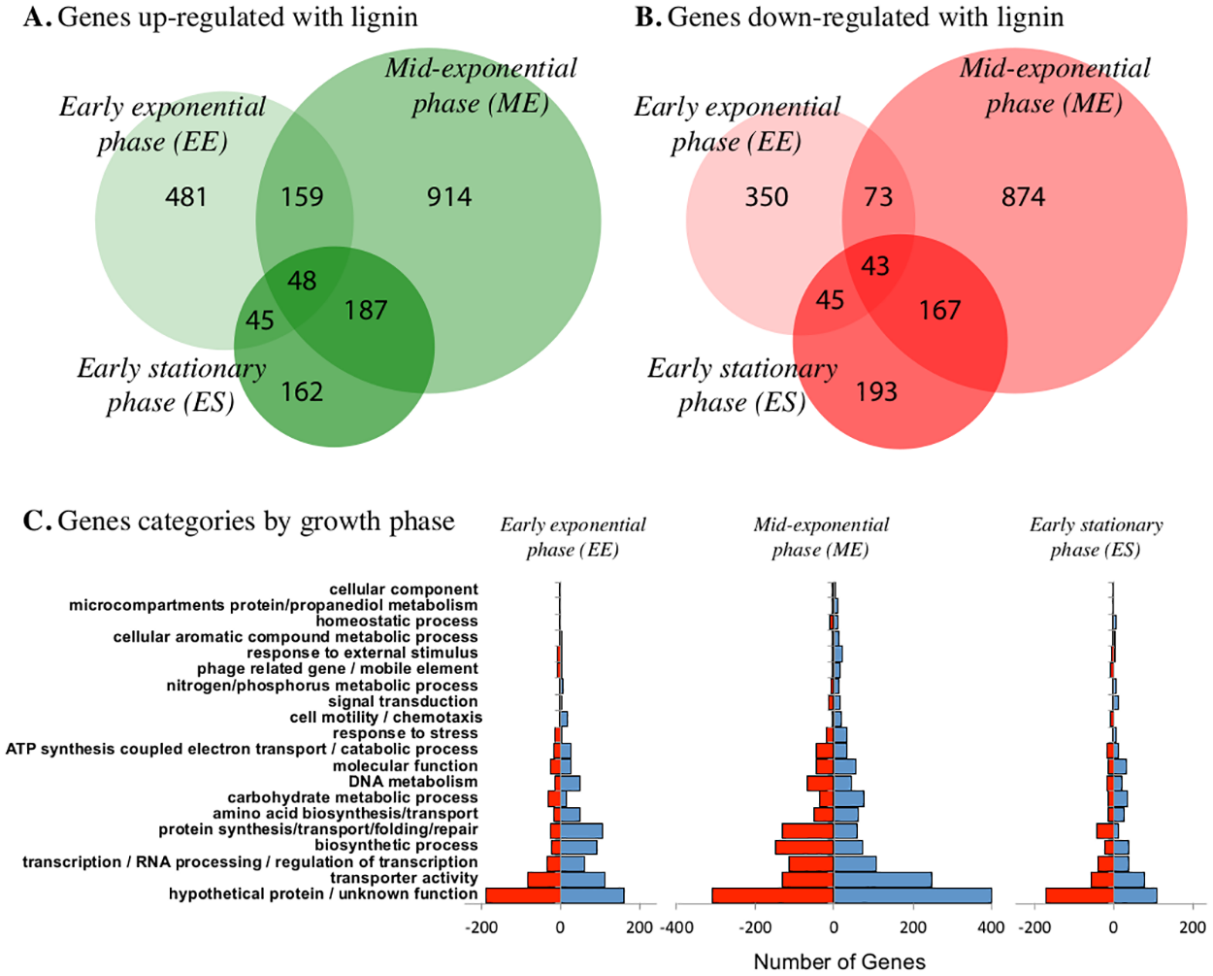
Venn diagrams of differentially expressed genes, where **(A)** green circles indicate number of total genes are up-regulated each time point, and **(B)** red circles indicate number of total genes were down-regulated each time point. EE indicates cells analyzed in early exponential phase, ME, mid-exponential phase and ES, early stationary phase, where actual times of sampling each cell population is indicated in the methods as well as in Fig 1A. **(C)** Changes in the gene profile as a result of lignin exposure in early exponential phase, mid-exponential and early stationary phase of growth. In the right side of each chart (blue), number of genes with increased relative abundance, and in the left side (red), number of genes with lower relative abundance. The genes are grouped according to functional class as defined by COG annotation.

One third (34%) of the 4445 predicted protein-encoding ORFs in the genome of SCF1 were differentially expressed at any time point during growth on glucose amended with lignin (mean 1533 genes +/- 275 standard error, Table 1). The highest number of differentially expressed genes was detected in mid-exponential phase (ME, 2465). Genes that were over expressed in more than a single time point represented ~17% of the total pool of over expressed genes, suggesting that although the degradation lignin is a dynamic process, there is a significant proportion of genes for whose differential abundance levels were sustained over time (Fig 2A and 2B).

**Table 1:** Total genes up and down regulated in each of the three points monitored during growth of SCF1 in lignin-amended cultures. The number in parenthesis indicates the percentage of the genes that were differentially expressed under each time.

Expression level	Time point			Total
	Early Exponential (EE)	Mid-Exponential (ME)	Early Stationary (ES)	
Up regulated	733	1308	442	2483
Down regulated	511	1157	448	2116
Total	1244 (27.9%)	2465 (55.4%)	890 (20%)	4599

Genes with differential expression in the presence of lignin were classified under 20 categories according to their annotation function in the genome (S1 Data). Genes encoding hypothetical protein and proteins with unknown function (667) had the highest number of genes with greater abundance under lignin-amended growth, followed by genes encoding proteins with transporter activity (436). Genes involved in transcription and RNA metabolism (206), biosynthesis (201) and protein synthesis (180) were also represented in abundance in the differential expression data (Fig 2C).

**Fig 3:**
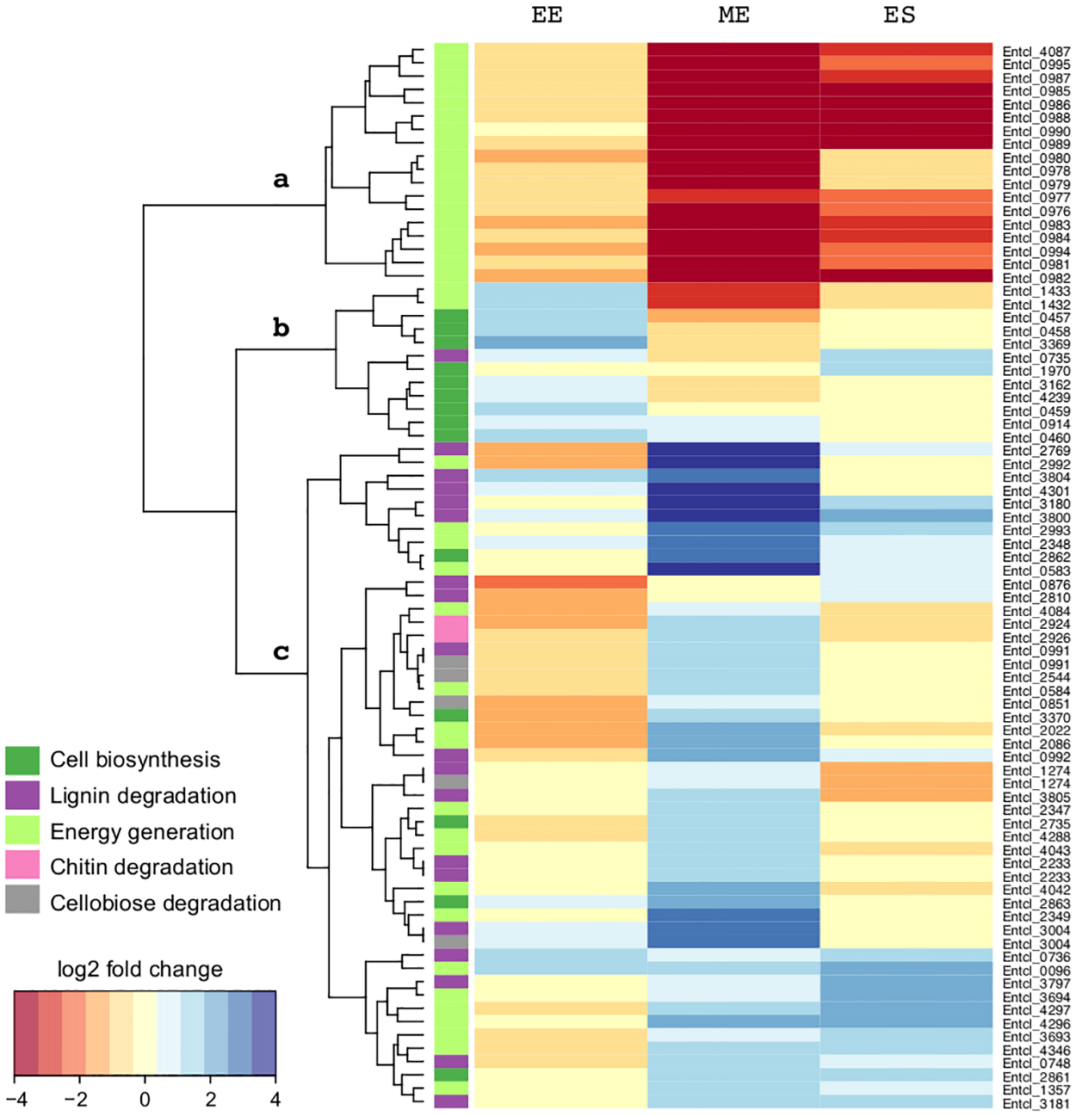
Clustering of transcripts shows response to lignin in genes of interest in *Enterobacter lignolyticus* SCF1 grown in glucose with and without lignin. Fold change indicates change in relative abundance of transcript in lignin-amended compared to unamended growth. Colors to the left of the heatmap indicate functional annotations as indicated. Each row represents gene expression of cells grown in lignin and glucose, compared to cells grown in glucose alone at the same time point.

Transcripts reflect the progression of activities associated with enhanced batch culture growth in the presence of lignin (Fig 3). Transcript production in response to lignin during early exponential phase growth was characterized by genes associated with biosynthesis and cell division. Catalase/peroxidase transcripts were over-expressed in lignin-amended compared to unamended conditions in every growth phase, but originating from different genes. These results suggest that lignin degradation is a very dynamic process that includes a wide set of genes involved in many cellular functions and takes place during the beginning of growth (early exponential) and the beginning of stationary phase, when the uptake of other substrates has stopped. By early stationary phase, waste products begin to accumulate as 60% of lignin has been degraded, and genes associated with continued expression of catalase/peroxidase enzymes and propanediol utilization related genes were found to be upregulated. The following sections describe in more detail the transcripts characterized by each growth phase.

### Early exponential phase growth in lignin is characterized by accelerated growth and signaling

During early exponential phase, many genes controlling growth and biosynthesis of new cell material were up-regulated in the presence of lignin (Fig 2C, S2 Table). The large and small subunits ribosomal RNAs, the rod shape-determining operon, the membrane-bound lytic murein transglycosylase, genes involved in the bacterial outer membrane biogenesis, chromosome partitioning and cell division processes were all significantly upregulated when lignin was present compared to lignin unamended conditions.

Lignin degradation rates are constrained by the complexity of the substrate, and most bacterial strains regulate their metabolic pathways for utilization of carbon sources by carbon catabolite repression [31]. This process allows mixed sugars to be utilized only sequentially, and gene regulation attributed to this process was observed in SCF1 growth in the presence of lignin. Many genes related to transporters were found to be down-regulated when glucose was available in the medium, but they were switched on after glucose was no longer metabolized (S3 Table). Genes belonging to the phosphoenolpyruvate phosphotransferase system (PTS) related to the uptake of mannose (Entcl_3690, Entcl_3691, Entcl_3692, Entcl_3811 and Entcl_3812), xylose (Entcl_0175 and Entcl_0176) and ribose (Entcl_0174, Entcl_1204, Entcl_1206, Entcl_4081) were down-regulated in early exponential phase. However, after glucose was made unavailable due to concentrations dipping below its half-saturation constant for uptake, they were transcribed in higher abundance.

The initial down regulation of genes related to the uptake of other sugars was also observed in the case of transcripts associated with degradation of cellobiose (Entcl_2546 and Entcl_3764), chitobiose (Entcl_2547 and Entcl_2548), maltose (Entcl_3261) and mannose (Entcl_4032, Entcl_4033, Entcl_4034 and Entcl_4036). Furthermore, many genes involved in the uptake of other sugars beside glucose were up-regulated after glucose uptake was stopped (Fig 2C) (Entcl_0166, Entcl_0167, Entcl_1205, Entcl_1207, Entcl_3382, Entcl_3383, Entcl_4082, Entcl_4174, and Entcl_4403, S4 Table). This was presumably a response to the lack of availability of glucose, an effect that has been observed in other fermentative anaerobes [32].

*Enterobacteriaceae* is a family of Gram negative bacteria originally described as part of the gut microbiota. As being isolated from Puerto Rico tropical forest soils [23], SCF-1 may harbor metabolic capabilities toward survival in more oligotrophic environments, including environment-sensing mechanisms coupled to extra- and intra-cellular signal transduction pathways. For instance, SCF-1 has an overrepresentation of genes related to two-component regulatory system compared to those members of the *Enterobacter* genus that resides in the gut or plant microbiota (Table 2). Among those, transcripts of the three genes encoding the UhpABC system (encoded by the Entcl_0034-Entcl_0037 operon), a two-component response regulator involved in the transport of hexoses as well as the transcripts of hexose phosphate transporter, UhpT (encoded by Entcl_0038), were more abundant in early exponential growth with lignin [33]. Other components of the two-component regulatory system were also up-regulated, including the sensory histidine kinase QseC (encoded by Entcl_0059), the osmolarity sensory histidine kinase, EnvZ (encoded by Entcl_0331), the sensory histidine kinase, QseC (encoded by Entcl_0729), the hybrid sensory histidine kinase, EvgA (encoded by Entcl_1061), the sensory histidine kinase, AtoS (encoded by Entcl_1498), and the Osmosensitive K+ channel histidine kinase, KdpD (encoded by Entcl_3122). Most of those genes highlight the challenge that SCF-1 faces when is surrounded with environments where lignin is present, even at small concentrations.

**Table 2:** Summary of genome attributes of *Enterobacter lignolyticus* SCF1 and three close relatives, *E*. *aerogenes*, *E*. *cloacae* ATCC13047, and *Enterobacter* sp. 638.

	*Enterobacter aerogenes*	*Enterobacter cloacae* ATCC13047	*Enterobacter sp* 638	*Enterobacter lignolyticus* SCF1
Class	Gamma-proteobacteria	Gamma-proteobacteria	Gamma-proteobacteria	Gamma-proteobacteria
Mode of life	gut microbiota /opportunistic bacteria	gut microbiota /opportunistic bacteria	Endophytic bacteria	Tropical soil bacterium
Type of metabolism	facultative anaerobic	facultative anaerobic	aerobic	facultative aerobe; grows well under completely oxic and anoxic conditions
Size of the genome (Mb)	5.27	5.59	4.67	4.81
Genes related to two-component regulatory system	21	20	27	33
Genes related to secretion systems	27	58	13	26
References	[71]	[71]	[72]	[23]

Many genes encoding proteins involved in resistance to environmental stresses were up-regulated in the presence of lignin, including the starvation sensing protein RspA (Entcl_1608), the starvation lipoprotein Slp paralog (Entcl_1986) and the universal stress protein family 3 (Entcl_2311). Carbon starvation protein A paralog (Entcl_3779) was also up-regulated in lignin-amended cultures after 7 hours, in agreement with former studies which found cstA to be upregulated at the protein and mRNA levels [20]. CstA, is a protein involved in escaping from starvation during nutrient scavenging and transition to stationary-phase growth [34] and has been shown to be regulated by the cyclic AMP (cAMP)-cAMP receptor protein complex, a global regulator involved in sugar metabolism [35]. Although the specific role of CstA in the metabolism of aromatic compounds remains to be elucidated, it has been demonstrated that carbon starvation proteins are induced by different aromatic pollutants [36], suggesting its involvement as a mechanism of adaptive response to the presence of a more recalcitrant carbon source [37]. Similar to *cstA*, the gene encoding the competence protein F (Entcl_0322) was found to be upregulated in lignin-amended cultures after 7 hours. This protein has also been shown to be part of the physiological response towards nutrient depravation, conferring cells the ability of uptake exogenous DNA and use it as a source of carbon and energy, providing an advantage during competition in nutrient limiting environments [38].

Within their wide set of tools to survive to dynamic environments, bacteria have evolved a way to respond to chemical or physical changes by modulating their behavior through gene expression. In one of those mechanisms, coined as "quorum sensing", bacteria respond to the concentration of a chemical molecule that reflects the bacterial population density allowing multicellular communication within a population [39]. One autoinducer, called AI-2, has been shown to be produced and recognized by several species allowing both intra and inter-cellular coordination [40]. We found that many genes related to the lsr operon (Entcl_0612, Entcl_0614, Entcl_0618 and Entcl_0619), the operon responsible for the uptake and processing of AI-2, were up-regulated in the presence of lignin.

### Mid-exponential samples capture switch towards degradation of lignin

The degradation of lignin is a dynamic process, which was evident during mid-exponential phase of growth where lignin amended culture transcripts reflected a decrease in growth rate likely caused by a switch in metabolic growth from only consuming glucose to depolymerization of lignin (Fig 1). During this period, many genes encoding for the large subunits of ribosomal RNAs were in lower abundance compared to the high abundance detected in early exponential phase (S5 Table), likely due to the switch towards lignin degradation.

Lignin degrading bacteria must overcome three main challenges towards efficient utilization of lignin as substrate. First, the mechanism of degradation should be oxidative [41] Secondly, since lignin is mainly composed by large highly insoluble molecules, lignin depolymerization enzymatic machinery has to be extracellular, or having an extracellular mediator. Third, the systems must provide a pool of enzymes with low level of specificity since lignin stereochemistry is highly variable [41]. For the sake of simplification, bacterial lignin degradation could be divided in two stages. Initially, depolymerization releases lignin monomers from the complex lignin structure mainly by the cleavage of ether linkages [9, 35]. After monomers are released, enzymes in peripheral pathways proceed with the breakdown of these monomers into common intermediates that are further processed by the central metabolism [42].

The genome of SCF1 contains several genes encoding for enzymes that have been previously implicated in the degradation of lignin [20, 23]. Glycoside hydrolases are common carbohydrate-active enzymes involved in plant biomass degradation, capable of hydrolyzing the glycosidic bonds between carbohydrates or between carbohydrates and lignin [43]. The genome of SCF-1 has four homologs encoding glycoside hydrolase family 1 β-glucosidases. Three of them (Entcl_0991, Entcl_1274 and Entcl_3004) were expressed in higher abundance during mid-exponential growth of lignin-amended cultures (Table 3). The 6-phospho-beta-glucosidase (Entcl_0991) is located in the same operon as the cellobiose-specific form of the phosphotransferase system component (Entcl_0992), suggesting the existence of an operon devoted to the degradation of components of plant material. Previous studies have shown glycoside hydrolases were abundant in a soil-derived consortia adapted to recalcitrant carbon sources, such as complex plant polysaccharides and lignin, and also highly expressed in degradation of cellulose and hemicellulose by hindgut microbiota associated to termites [44, 45].

**Table 3:** Genes differentially regulated during growth related to lignin degradation. Differential expression was defined as transcripts with adjusted p-values <0.05 and absolute value of log2 fold change >1 for these comparisons.

Gene ID	Annotation	Gene name	Fold change in transcripts		
			EE	ME	ES
Entcl_1274	6-phospho-beta-glucosidase (EC 3.2.1.86)		0.041	0.991	-1.131
Entcl_3004	6-phospho-beta-glucosidase (EC 3.2.1.86)		0.437	2.680	-0.150
Entcl_0991	6-phospho-beta-glucosidase (EC 3.2.1.86)	ascB	-0.915	1.496	-0.362
Entcl_0992	PTS system, arbutin-, cellobiose-, and salicin-specific IIBC component (EC 2.7.1.69)		-0.653	2.291	0.463
Entcl_0735	putative laccase (EC 1.10.3.2)		0.523	-0.921	1.354
Entcl_0736	Probable Fe-S oxidoreductase family 2		1.516	1.037	1.106
Entcl_0748	Predicted oxidoreductases (related to aryl-alcohol dehydrogenases)		-0.626	1.684	0.883
Entcl_4301	Catalase (EC 1.11.1.6) / Peroxidase (EC 1.11.1.7)		0.560	3.932	0.146
Entcl_2769	Flavoprotein wrbA		-1.502	3.638	0.700
Entcl_3180	Alkyl hydroperoxide reductase protein F (EC 1.6.4.-)		0.186	3.513	1.719
Entcl_3181	Alkyl hydroperoxide reductase protein C (EC 1.6.4.-)		-0.010	1.468	1.126
Entcl_3797	4-hydroxyphenylacetate degradation bifunctional isomerase/decarboxylase, HpaG2 subunit		0.111	0.525	1.889
Entcl_3800	5-carboxymethyl-2-hydroxymuconate delta-isomerase (EC 5.3.3.10)		0.384	3.639	1.939
Entcl_3804	Transcriptional activator of 4-hydroxyphenylacetate 3-monooxygenase operon, XylS/AraC family		1.314	2.640	-0.058
Entcl_3805	4-hydroxyphenylacetate 3-monooxygenase (EC 1.14.13.3)		0.000	1.706	-1.529
Entcl_2233	Succinate-semialdehyde dehydrogenase [NADP+]		0.340	1.543	-0.248
Entcl_0876	Succinate-semialdehyde dehydrogenase [NADP+] (EC 1.2.1.16)		-1.962	0.231	0.408
Entcl_2810	Gamma-glutamyl-aminobutyraldehyde dehydrogenase (EC 1.2.1.-)		-1.292	0.011	0.519
Entcl_2233	Succinate-semialdehyde dehydrogenase [NADP+]		0.340	1.543	-0.248

During the degradation of lignin, many phenolic compounds are release into the medium. Those metabolites including acids (ferulic acid, vanillic acids and 4-hydroxybenzoic acid), alcohols (guaiacol and catechol, etc) and aldehydes (vanillin, 4-hydroxylbenzaldehyde, etc) have been reported to alter the permeability of cellular membranes at even small concentrations [46]. One of the strategies of microbes towards to cope with those stressful conditions is through the breakdown of phenols to nontoxic compounds. For instance, laccases (phenoloxidases, EC 1.10.3.2) are multicopper-containing enzymes capable of breaking phenols, aromatic and aliphatic C–C bonds by oxidation of phenolic units using molecular oxygen as the final electron acceptor [47, 48]. Although fungal laccases have been shown to play a key role during lignin degradation in the white-rot fungi [49–51], little is known about their function in their bacterial counterparts. Recent research has focused on the involvement of laccases during decomposition of lignin by strains of the soil-inhabiting microorganisms *Streptomyces*, where the deletion of the gene encoding a laccase partially impaired lignin degradation [52]. Previous evidence has suggested that the genome of SCF1 contains two putative laccases homolgs [20]. One of those genes, encoding a multicopper oxidase type 3 (Entcl _0735), was found to be up-regulated in the presence of lignin. Its adjacent gene (Entcl_0736) that seems to be co-transcribed with, correspond to an uncharacterized enzyme that contains Radical SAM domain protein using cobalamine (Vitamine B-12) as a cofactor. Several genes involved in the synthesis of cobalamine (Entcl_1738, Entcl_1762, Entcl_1763, Entcl_1764, Entcl_1765, Entcl_1766, Entcl_1767, Entcl_1768, and Entcl_1771) were found to be up-regulated in cultures were lignin was added. Furthermore, both laccases are predicted to be extracellular and periplasmic, respectively by PSORT [53] suggesting that they may be secreted. Under anaerobic growth conditions, the role laccases during lignin degradation remains questionable, due to the fact that those enzymes employ dioxygen as electron acceptor to carry out single electron oxidations of organic compounds. To this date, it is unclear how laccases could perform oxidation with an alternative electron acceptor. Recent evidence carried out with soil bacterium *Stenotrophomonas maltophilia* AAP56 found that laccase activity was highly dependent on the environment and it was greatly induced by anoxic conditions. More importantly, laccase activity is often induced by addition of CuSO_4_ [54]. In this example, laccase activity was greatly induced by the addition of 100 μM of CuSO_4_ in the γ-Proteobacterium JB, an alkali-tolerant soil isolate [55]. Therefore, as the trace element solution utilized in the growth medium has some Cu, and our lignin contains some sulfur impurities, we cannot rule out the possibility that laccases were induced by CuSO_4_, and not by lignin.

Aryl-alcohol dehydrogenases are enzymes capable of cleaving the β-aryl ether bonds, and have been shown to play a key role during depolymerization of lignin by white-rot fungi [56, 57]. As a part of this potential mechanism in SCF-1, we found that the transcripts of an aryl-alcohol dehydrogenases (Entcl_0748) were expressed in higher abundance in lignin-amended cultures (Table 3). Aryl-hydrogenases provide the hydrogen peroxide needed by ligninolytic high redox-potential peroxidases during fungal degradation of lignin [57]. Peroxidases aid in lignin degradation by removing one electron from the non-phenolic units of lignin [58]. Indeed, lignin peroxidases (EC 1.11.1.14) utilize hydrogen peroxide (H_2_O_2_) as the co-substrate in addition to a mediator, veratryl alcohol, to degrade lignin and other phenolic compounds. H_2_O_2_ is reduced to H_2_O by gaining an electron from the then oxidized LiP and then LiP release this electron to vetraryl alcohol that becomes aldehyde and gets reduced to its alcohol by gaining electrons from lignin [35]. The utilization of peroxidases in lignin degradation was previously hypothesized because SCF1 was isolated from the Luquillo LTER soils, where peroxidase activities were detected across a large area [20]. One peroxidase (encoded by Entcl_4301) and a flavoprotein disulfide reductase (encoded by Entcl_2769), an enzyme capable of sensing the local concentrations of hydrogen peroxide, were found to be up-regulated in the presence of lignin during mid-exponential phase, the period in which 60% of the lignin amended in the medium was utilized. Also, the genes encoding alkyl hydroperoxide reductases (encoded by Entcl_3180 and Entcl_3181), one of the main scavenger of H_2_O_2_ inside the cells, were found to be expressed in higher abundance in the presence of lignin, during the same sampling time. Although lignin peroxidases may be important enzymes for lignin degradation in SCF-1, the way in which hydrogen peroxide is provided at a constant rate under anaerobic conditions and its role during the anaerobic degradation of lignin remains unknown.

Another proposed mechanism for the degradation of lignin is via 4-hydroxyphenylacetate catabolic pathway. This pathway is composed by nine enzymes encoded by genes within a single genomic region (HpaRGEDFHIXABC) previously described as an aromatic degrading gene cluster. This cluster has been shown to be involved in the transformation of 4-hydroxyphenylacetate, mainly coming from the degradation of aromatic compounds, to intermediates of the central metabolism [23]. We found the gene encoding for the first gene of this pathway (Entcl_3797) to be up-regulated in the presence of lignin, suggesting that the pathway was turn on. Furthermore, three genes associated to this cluster (Entcl_3800, Entcl_3804 and Entcl_3805) were expressed in higher abundance when lignin was present. This pathway takes 4-hydroxyphenylacetate, a resulting compound from the fermentation of short-chain peptides and amino acids, and degrades it to pyruvate and succinate-semialdehyde [59, 60]. Because of its toxicity, succinate-semialdehyde has to be quickly converted to succinate via NADP^+^-dependent succinate semialdehyde dehydrogenase previous entering into the tricarboxylic acid cycle [61]. The genome of SCF-1 contains four homologs encoding for this enzyme (Entcl_2233, Entcl_2291, Entcl_0876 and Entcl_2810) and one of them (Entcl_2233) was expressed in higher abundance in lignin-amended cultures.

Other genes with a potential role during aromatic metabolism that support lignin degradation were found to be differentially expressed during lignin-amended growth. For instance, genes encoding for two anthranilate synthases (Entcl_2523 and Entcl_2524), a peripheral pathway that play an important role during anaerobic degradation of heterocyclic aromatic compounds, were found to be among top-5 upregulated genes in lignin amended cultures [42]. Also, genes encoding enzymes that belong to xanthine/uracil family permeases, such as Entcl_0083, Entcl_1586, Entcl_4435, Entcl_3516, Entcl_3019 and genes encoding for enzymes that catalyzes the further metabolization of xanthine, such as Entcl_4100, Entcl_3480, Entcl_2780, Entcl_2781, Entcl_2782, and Entcl_2795, were found to be up-regulated during lignin-amended mid-exponential growth. This set of enzymes belong to a mechanism of recycling of uric acid, xanthine and other nitrogenous products that has been previously reported to be relevant in the gut microbiome of wood-feeding beetles [62].

Lignin amendment was also associated with higher abundance of transcripts for genes associated with the uptake and metabolism of sugars. For example, the gene encoding for glucokinase (Entcl_1357), an enzyme that catalyzes the first step in glycolysis (reaction #1, S1 Fig), was found to be upregulated during mid-exponential growth, The expression of genes encoding for glucose-6-phosphate isomerase (Entcl_3693 and Entcl_3694) (reaction #2, S1 Fig), 6-phosphofructokinase class II (Entcl_2086 and Entcl_4346) (reaction #3, S1 Fig) and fructose-1,6-bisphosphatase, GlpX type (Entcl_0096) (reaction #4, S1 Fig) were also found to be expressed in higher abundance in early stationary phase. Similar behavior was observed in the genes encoding for the steps downstream in glycolysis, such as fructose-bisphosphate aldolase, class II (encoded by Entcl_4084) (reaction #5, S1 Fig), and the NAD-dependent glyceraldehyde-3-phosphate dehydrogenase (encoded by Entcl_2022).

### Lignin-amended cultures produce very different metabolites by early-exponential phase

*E*. *lignolyticus* SCF1, as other enterobacteria, primarily carry out heteroenzymatic fermentation using pentose and hexose sugars as carbon and energy sources, and producing acetate, ethanol, formate and lactate, as main fermentation products [23]. A high concentration level of glucose remained in the medium after SCF-1 stopped growing, 45 mM in the presence of lignin and 40 mM in the control. The inability to keep metabolizing the substrates can be explained by the increased formation of acidic metabolites, such as acetate, formate, lactate, and succinate, which destroys the cell's ability to maintain internal pH [63].

Lignin amendment resulted in a significant difference in the production of fermentation subproducts, which may be attributed to the higher quantity of C atoms that have to be funneled into common intermediates of the central metabolism (pyruvate and Acetyl-CoA). For instance, the pyruvate formate-lyase, an enzyme in charge of supplying the citric acid cycle with acetyl-CoA during anaerobic glycolysis (encoded by Entcl_0584, Entcl_2993, Entcl_4043, Entcl_4296, Entcl_4297) and also its activating enzyme (encoded by Entcl_2992, Entcl_4042, and Entcl_4288; S6 Table) were expressed in higher abundance when lignin degradation rate was at its maximum. There was about 5 mM of more formate that was released by the cells in lignin-amended cultures (Fig 1C), though most of this accumulated in the first 15 hours of growth.

Many genes belonging to metabolic pathways devoted to degrade other components of plant material were also regulated under lignin amendments. For instance, several genes encoding enzymes involved in the degradation of pectin, a structural polysaccharide that is key for the stability of plant cell walls, were found to be upregulated (S7 Table). A considerable high number of genes (48) remained upregulated across the three growth phases underlying the idea that some of those genes may represent a set of metabolic functions that have to be constitutively expressed in the presence of lignin, rather than a part of a progressive metabolic or biological mechanism towards degradation of lignin (S8 Table).

Because of the increased formate production and transcription of formate hydrogenlyase (FHL) complex transcripts with lignin, we hypothesized that SCF-1 would produce hydrogen as an alternative to regulate pH in the lignin-amended treatments. Many members of *Enterobacteriaceae*, such as *E*.*coli* and *Salmonella enterica*, contain a membrane-associated FHL complex capable to utilize formic acid as electron donor and protons as electron acceptors during the production of H_2_ and CO_2_ [64, 65]. This complex allows the accumulation of the excess of reducing equivalents produced by fermentation into hydrogen, preventing further acidification of the medium and promoting interspecies H_2_ transfer. The FHL complex is composed of two main parts that catalyze hydrogen production from formate in two steps. The molybdenum-dependent formate dehydrogenase (FDHH) encoded by fdhF gene (Entcl_4087) catalyzes the reversible oxidation of formate to CO_2_ with the release of protons and two electrons. A second step is catalyzed by the NiFe hydrogenase, Hyd3, encoded by Entcl_0986, that utilize these electrons to further reduce protons to H_2_ [66]. Hyd3, belongs to the Group 4 [NiFe] hydrogenases, a phylogenetically distinct family of membrane-bound, cytoplasmically oriented hydrogenases that so far has been rarely characterized, and are predominantly involved in H_2_ production rather than H_2_ oxidation [67, 68]. Hyd3 differs from other hydrogenases because it allows accumulation of significant concentrations H_2_, without inhibition, making SCF-1 especially well-suited to biotechnological H_2_ production.

Cell suspensions were capable of anaerobically producing H_2_ in the presence of formate (150 mM) and lignin (0.05% w/w) (S4 Fig), which is evidence suggesting that the FHL complex is functional in *E*. *lignolyticus* SCF-1. However, during hydrogen production in cell suspensions, ~30% less H_2_ was produced in those incubations where lignin was added compared to formate added alone. In the RNA-seq results, the two components of formate hydrogenlyase complex, the formate dehydrogenase H (Entcl_4087), the seven subunits of the formate hydrogenlyase complex (Entcl_0982-Entcl_0988), its transcriptional activator (Entcl_0976) and the neighbor NiFe hydrogenase assembly enzymes (Entcl_0977-Entcl_0982) were down-regulated during the late stages of growth. On the other hand, genes belonging to the formate dehydrogenase type N (Entcl_2347–2349), an enzyme that generally oxidizes formate taking electrons towards dissimilatory nitrate reduction, were expressed in higher abundance when lignin was present after mid-exponential growth. We also observed a higher concentration of acetate in those amended cultures (Fig 1C). This overproduction could be attributed to the higher expression of Phosphate acetyltransferase (Entcl_1432) (reaction number 21, S1 Fig) and acetate kinase (Entcl_0583 and Entcl_1433) (reaction number 22, S1 Fig) in the earlier stages of growth.

## Conclusions

Several omic techniques (genomics, transcriptomics, proteomics and metabolomics) are suitable to improve our understanding of microbial communities, enzyme interactions, and how lignocellulose breakdown occurs, in both isolated cultures or microbial communities [69, 70]. Although, there is current limited understanding of how aromatic metabolism is capable to support anaerobic lignin degradation, the set of up-regulated enzymes during lignin-amended growth could conceivably suggest their role during this process. By examining the degradation of lignin in batch culture over time, we were able to capture the transcripts involved in the initial accelerated growth and transport of glucose in the presence of lignin during early exponential phase, the diverse complement of lignin-degrading enzymes expressed during mid-exponential growth, and transporters and metabolic machinery responsible for the increased formate production observed concurrent with 60% of lignin reduced. This work will contribute to our understanding of anaerobic lignin degradation as well as the cellular machinery necessary to balance metabolism and manage toxic products formed as a result of growth in lignin amended media.

## Supporting information

S1 Data Fold change in ratios of transcripts of genes significantly differentially expressed in lignin compared to unamended growth in glucose. Positive numbers indicate upregulation and negative numbers indicate downregulation. T1 means beginning of exponential phase, T2 means mid-exponential phase, and T3 means beginning of stationary phase.
(XLSX)

S1 Fig Reconstruction of main metabolic pathways of glucose and xylose metabolism. Red and blue boxes indicate the consumption and production of the compound, respectively. Numbers in parenthesis indicate the name of the reaction as following: (1)ATP:D-glucose 6-phosphotransferase, EC 2.7.1.2, encoded by Entcl_1357; (2) Glucose-6-phosphate isomerase, EC 5.3.1.9, encoded by Entcl_3693, Entcl_4141; (3) ATP:D-fructose-6-phosphate-1-phosphotransferase, EC2.7.1.11, encoded by Entcl_2086 and Entcl_4346; (4) D-Fructose 1,6 biphosphate-1-phosphohydrolase, EC 3.1.3.11, encoded by Entcl_3933; (5) D-fructose 1,6 biphosphate D-glyceraldehyde 3-phosphate lyase, EC 4.1.2.13, encoded by Entcl_0829; (6) D-glyceraldehide-3-phosphate ketol-isomerase, EC 5.3.1.1, encoded by Entcl_0092; (7) glyceraldehyde-3-phosphate dehydrogenase, EC 1.2.1.12, encoded by Entcl_2214, Entcl_2615, and Entcl_2022; (8) phosphoglycerate kinase, EC 2.7.2.3, encoded by Entcl_0828; (9) phosphoglyceratemutase, EC 5.4.2.11, encoded by Entcl_3728, Entcl_0131, and Entcl_3075; (10) 2-phospho-D-glycerate hydro-lyase, EC 4.2.1.11, encoded by Entcl_0935; (11) phosphoenolpyruvatecarboxykinase (ATP), EC 4.1.1.49 encoded by Entcl_0332; (12) malate dehydrogenase, EC 1.1.1.37 encoded by Entcl_0474; (13) fumaratehydratase, EC 4.2.1.2, encoded by Entcl_1727, Entcl_1688, Entcl_2213, Entcl_2234; (14) succinate dehydrogenase, EC 1.3.5.1, encoded by Entcl_3102, Entcl_3103, Entcl_3104, and Entcl_3105; (15) phosphoenolpyruvate kinase, EC 2.7.1.40, encoded by Entcl_1936 and Entcl_2127; (16) Pyruvate-formatelyase, EC 2.3.1.54, encoded by Entcl_2993, Entcl_4043, Entcl_4289, Entcl_4296; Formatehydrogenlyase complex, encoded by Entcl_0982, Entcl_0983, Entcl_0985, Entcl_0986, Entcl_0987, and Entcl_0989; (18) pyruvate ferredoxin/flavodoxinoxidoreductase, EC 1.2.7.1, encoded by Entcl_2436; (19) L-lactate dehydrogenase, EC:1.1.1.28, encoded by Entcl_1569 and Entcl_3787; (20) Acetolactate synthase, EC 2.2.1.6, encoded by Entcl_0028, Entcl_3647, Entcl_3648, Entcl_4268, and Entcl_4269; (21) Phosphate acetyltransferase, EC 2.3.1.8, encoded by Entcl_1309, and Entcl_1432; (22) Acetate kinase, EC 2.7.2.1, encoded by Entcl_0583, Entcl_1740, and Entcl_1433; (23) Acetaldehyde dehydrogenase, EC 1.2.1.10, encoded by Entcl_2072, and Entcl_1745; (24) Alcohol dehydrogenase, EC 1.1.1.1, encoded by Entcl_2072, Entcl_0739, Entcl_1314, Entcl_1745, Entcl_3458, and Entcl_4294; (25) Xylose Isomerase, EC 5.3.1.5, encoded by Entcl_0177; (26) Xylulose kinase, EC 2.7.1.17, encoded by Entcl_0178; (27) L-ribulose-5-phosphate 4-epimerase, EC 5.1.3.4, encoded by Entcl_0156, Entcl_3664, Entcl_3966; (28) Phosphogluconate dehydrogenase, EC 1.1.1.44, encoded by Entcl_1664; (29) Gluconokinase, EC 2.7.1.12, encoded by Entcl_0304, and Entcl_3682; (30) Gluconolactonase, EC 3.1.1.17, encoded by Entcl_0174; and (31) 2-dehydro-3-deoxy-phosphogluconate aldolase, EC 4.1.2.14, encoded by Entcl_1940.
(DOCX)

S2 Fig Optical density measured at 600 nm (OD_600_) as a metric of changing cell biomass over time under lignin-amended (filled circles) versus unamended (open circles) growth conditions of *Enterobacter lignolyticus* SCF1. We systematically found that the optical density of lignin-amended treatments were higher that unamended controls.
(DOCX)

S3 Fig Colony forming units (CFUs) as a metric of changing cell biomass over time under lignin-amended (filled circles) versus unamended (open circles) growth conditions of *Enterobacter lignolyticus* SCF1. (DOCX)

S4 Fig Cell suspensions of SCF1 were introduced to minimal media amended with no carbon source (control, open circles), 150 mM formate (formate, closed squares), or 150 mM formate and 0.05% lignin (formate+lignin, open diamonds). Cell suspensions were monitored over time for headspace accumulation of hydrogen gas (H_2_), which should have been produced as a result of formatehydrogenlyase activity.
(DOCX)

S1 Table Summary of statistics of cell density as measured by optical density (OD600) and colony forming units (CFUs) for time points showing significant effect of lignin addition. (DOCX)

S2 Table Genes differentially expressed in early exponential (EE) phase during growth of SCF1 on lignin-amended versus unamended growth. Differential expression was defined as transcripts with adjusted p-values <0.05 and absolute value of log2 fold change >1 for these comparisons.
(DOCX)

S3 Table Genes differentially regulated during growth related to the catabolism of sugars. Differential expression was defined as transcripts with adjusted p-values <0.05 and absolute value of log2 fold change >1 for these comparisons.
(DOCX)

S4 Table Genes differential expressed during growth of SCF1 on lignin-amended versus unamended growth involved in the uptake of other sugars beside glucose. Differential expression was defined as transcripts with adjusted p-values <0.05 and absolute value of log2 fold change >1 for these comparisons.
(DOCX)

S5 Table Genes differentially expressed during growth of SCF1 on lignin-amended versus unamended growth. Differential expression was defined as transcripts with adjusted p-values <0.05 and absolute value of log2 fold change >1 for these comparisons.
(DOCX)

S6 Table Genes differentially regulated during growth related to energy generation. Differential expression was defined as transcripts with adjusted p-values <0.05 and absolute value of log2 fold change >1 for these comparisons.
(DOCX)

S7 Table Genes differentially regulated during growth related to other metabolic pathways. Differential expression was defined as transcripts with adjusted p-values <0.05 and absolute value of log2 fold change >1 for these comparisons.
(DOCX)

S8 Table Genes differentially regulated along three measured growth phases. Differential expression was defined as transcripts with adjusted p-values <0.05 and absolute value of log2 fold change >1 for these comparisons.
(DOCX)
