# Supplementary material for: Multi-time series RNA-seq analysis of Enterobacter lignolyticus SCF1 during growth in lignin-amended medium
Source: PLoS One. 2017 Oct 19;12(10):e0186440. doi: 10.1371/journal.pone.0186440 (PMC5648182; doi:10.1371/journal.pone.0186440)
Supplement: S1 Fig — Red and blue boxes indicate the consumption and production of the compound, respectively. Numbers in parenthesis indicate the name of the reaction as following: (1)ATP:D-glucose 6-phosphotransferase, EC 2.7.1.2, encoded by Entcl_1357; (2) Glucose-6-phosphate isomerase, EC 5.3.1.9, encoded by Entcl_3693, Entcl_4141; (3) ATP:D-fructose-6-phosphate-1-phosphotransferase, EC2.7.1.11, encoded by Entcl_2086 and Entcl_4346; (4) D-Fructose 1,6 biphosphate-1-phosphohydrolase, EC 3.1.3.11, encoded by Entcl_3933; (5) D-fructose 1,6 biphosphate D-glyceraldehyde 3-phosphate lyase, EC 4.1.2.13, encoded by Entcl_0829; (6) D-glyceraldehide-3-phosphate ketol-isomerase, EC 5.3.1.1, encoded by Entcl_0092; (7) glyceraldehyde-3-phosphate dehydrogenase, EC 1.2.1.12, encoded by Entcl_2214, Entcl_2615, and Entcl_2022; (8) phosphoglycerate kinase, EC 2.7.2.3, encoded by Entcl_0828; (9) phosphoglyceratemutase, EC 5.4.2.11, encoded by Entcl_3728, Entcl_0131, and Entcl_3075; (10) 2-phospho-D-glycerate hydro-lyase, EC 4.2.1.11, encoded by Entcl_0935; (11) phosphoenolpyruvatecarboxykinase (ATP), EC 4.1.1.49 encoded by Entcl_0332; (12) malate dehydrogenase, EC 1.1.1.37 encoded by Entcl_0474; (13) fumaratehydratase, EC 4.2.1.2, encoded by Entcl_1727, Entcl_1688, Entcl_2213, Entcl_2234; (14) succinate dehydrogenase, EC 1.3.5.1, encoded by Entcl_3102, Entcl_3103, Entcl_3104, and Entcl_3105; (15) phosphoenolpyruvate kinase, EC 2.7.1.40, encoded by Entcl_1936 and Entcl_2127; (16) Pyruvate-formatelyase, EC 2.3.1.54, encoded by Entcl_2993, Entcl_4043, Entcl_4289, Entcl_4296; Formatehydrogenlyase complex, encoded by Entcl_0982, Entcl_0983, Entcl_0985, Entcl_0986, Entcl_0987, and Entcl_0989; (18) pyruvate ferredoxin/flavodoxinoxidoreductase, EC 1.2.7.1, encoded by Entcl_2436; (19) L-lactate dehydrogenase, EC:1.1.1.28, encoded by Entcl_1569 and Entcl_3787; (20) Acetolactate synthase, EC 2.2.1.6, encoded by Entcl_0028, Entcl_3647, Entcl_3648, Entcl_4268, and Entcl_4269; (21) Phosphate acetyltransferase, EC 2 [file pone.0186440.s002.docx]

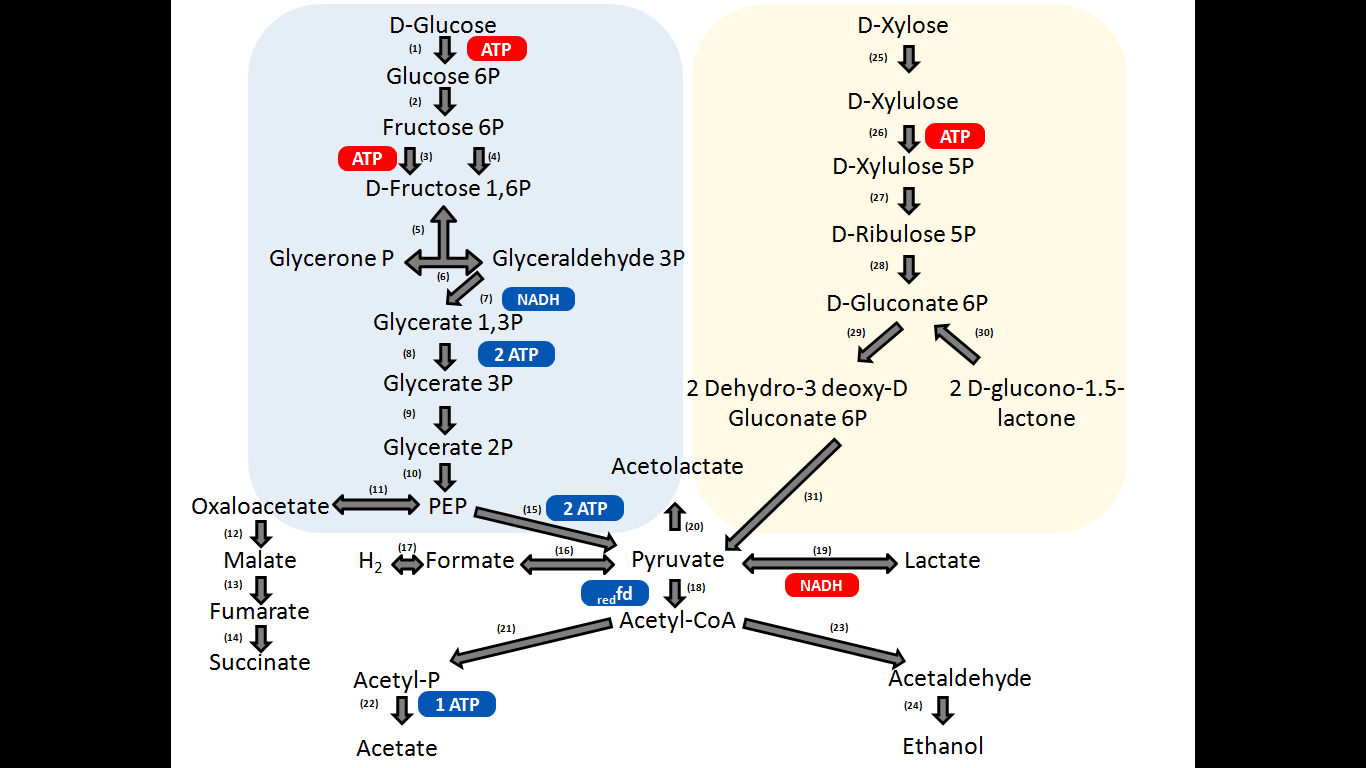


**S1 Figure.** Reconstruction of main metabolic pathways of Glucose and Xylose metabolism. Red and blue boxes indicate the consumption and production of the compound, respectively. Numbers in parenthesis indicate the name of the reaction as following: (1)ATP:D-glucose 6-phosphotransferase, EC 2.7.1.2, encoded by Entcl_1357; (2) Glucose-6-phosphate isomerase, EC 5.3.1.9, encoded by Entcl_3693, Entcl_4141; (3) ATP:D-fructose-6-phosphate-1-phosphotransferase, EC2.7.1.11, encoded by Entcl_2086 and Entcl_4346; (4) D-Fructose 1,6 biphosphate-1-phosphohydrolase, EC 3.1.3.11, encoded by Entcl_3933; (5) D-fructose 1,6 biphosphate D-glyceraldehyde 3-phosphate lyase, EC 4.1.2.13, encoded by Entcl_0829; (6) D-glyceraldehide-3-phosphate ketol-isomerase, EC 5.3.1.1, encoded by Entcl_0092; (7) glyceraldehyde-3-phosphate dehydrogenase, EC 1.2.1.12, encoded by Entcl_2214, Entcl_2615, and Entcl_2022; (8) phosphoglycerate kinase, EC 2.7.2.3, encoded by Entcl_0828; (9) phosphoglyceratemutase, EC 5.4.2.11, encoded by Entcl_3728, Entcl_0131, and Entcl_3075; (10) 2-phospho-D-glycerate hydro-lyase, EC 4.2.1.11 , encoded by Entcl_0935; (11) phosphoenolpyruvatecarboxykinase (ATP), EC 4.1.1.49 encoded by Entcl_0332; (12) malate dehydrogenase, EC 1.1.1.37 encoded by Entcl_0474; (13) fumaratehydratase, EC 4.2.1.2, encoded by Entcl_1727, Entcl_1688, Entcl_2213, Entcl_2234; (14) succinate dehydrogenase, EC 1.3.5.1, encoded by Entcl_3102, Entcl_3103, Entcl_3104, and Entcl_3105; (15) phosphoenolpyruvate kinase, EC 2.7.1.40, encoded by Entcl_1936 and Entcl_2127; (16) Pyruvate-formatelyase, EC 2.3.1.54, encoded by Entcl_2993, Entcl_4043, Entcl_4289, Entcl_4296; Formatehydrogenlyase complex, encoded by Entcl_0982, Entcl_0983, Entcl_0985, Entcl_0986, Entcl_0987, and Entcl_0989; (18) pyruvate ferredoxin/flavodoxinoxidoreductase, EC 1.2.7.1, encoded by Entcl_2436; (19) L-lactate dehydrogenase, EC:1.1.1.28, encoded by Entcl_1569 and Entcl_3787; (20) Acetolactate synthase, EC 2.2.1.6, encoded by Entcl_0028, Entcl_3647, Entcl_3648, Entcl_4268, and Entcl_4269; (21) Phosphate acetyltransferase, EC 2.3.1.8, encoded by Entcl_1309, and Entcl_1432; (22) Acetate kinase, EC 2.7.2.1, encoded by Entcl_0583, Entcl_1740, and Entcl_1433; (23) Acetaldehyde dehydrogenase, EC 1.2.1.10, encoded by Entcl_2072, and Entcl_1745; (24) Alcohol dehydrogenase, EC 1.1.1.1, encoded by Entcl_2072, Entcl_0739, Entcl_1314, Entcl_1745, Entcl_3458, and Entcl_4294; (25) Xylose Isomerase, EC 5.3.1.5, encoded by Entcl_0177; (26) Xylulose kinase, EC 2.7.1.17, encoded by Entcl_0178; (27) L-ribulose-5-phosphate 4-epimerase, EC 5.1.3.4, encoded by Entcl_0156, Entcl_3664, Entcl_3966; (28) Phosphogluconate dehydrogenase, EC 1.1.1.44, encoded by Entcl_1664; (29) Gluconokinase, EC 2.7.1.12, encoded by Entcl_0304, and Entcl_3682; (30) Gluconolactonase, EC 3.1.1.17, encoded by Entcl_0174; and (31) 2-dehydro-3-deoxy-phosphogluconate aldolase, EC 4.1.2.14 , encoded by Entcl_1940.
