# Supplementary material for: Multi-time series RNA-seq analysis of Enterobacter lignolyticus SCF1 during growth in lignin-amended medium
Source: PLoS One. 2017 Oct 19;12(10):e0186440. doi: 10.1371/journal.pone.0186440 (PMC5648182; doi:10.1371/journal.pone.0186440)
Supplement: S2 Fig — We systematically found that the optical density of lignin-amended treatments were higher that unamended controls. (DOCX) [file pone.0186440.s003.docx]

**S2 Figure**. Optical density measured at 600 nm (OD_600_) as a metric of changing cell biomass over time under lignin-amended (filled circles) versus unamended (open circles) growth conditions of *Enterobacter lignolyticus* SCF1. We systematically found that the optical density of lignin-amended treatments were higher that unamended controls.
