# Supplementary material for: Multi-time series RNA-seq analysis of Enterobacter lignolyticus SCF1 during growth in lignin-amended medium
Source: PLoS One. 2017 Oct 19;12(10):e0186440. doi: 10.1371/journal.pone.0186440 (PMC5648182; doi:10.1371/journal.pone.0186440)
Supplement: S4 Fig — Cell suspensions were monitored over time for headspace accumulation of hydrogen gas (H2), which should have been produced as a result of formatehydrogenlyase activity. (DOCX) [file pone.0186440.s005.docx]

**
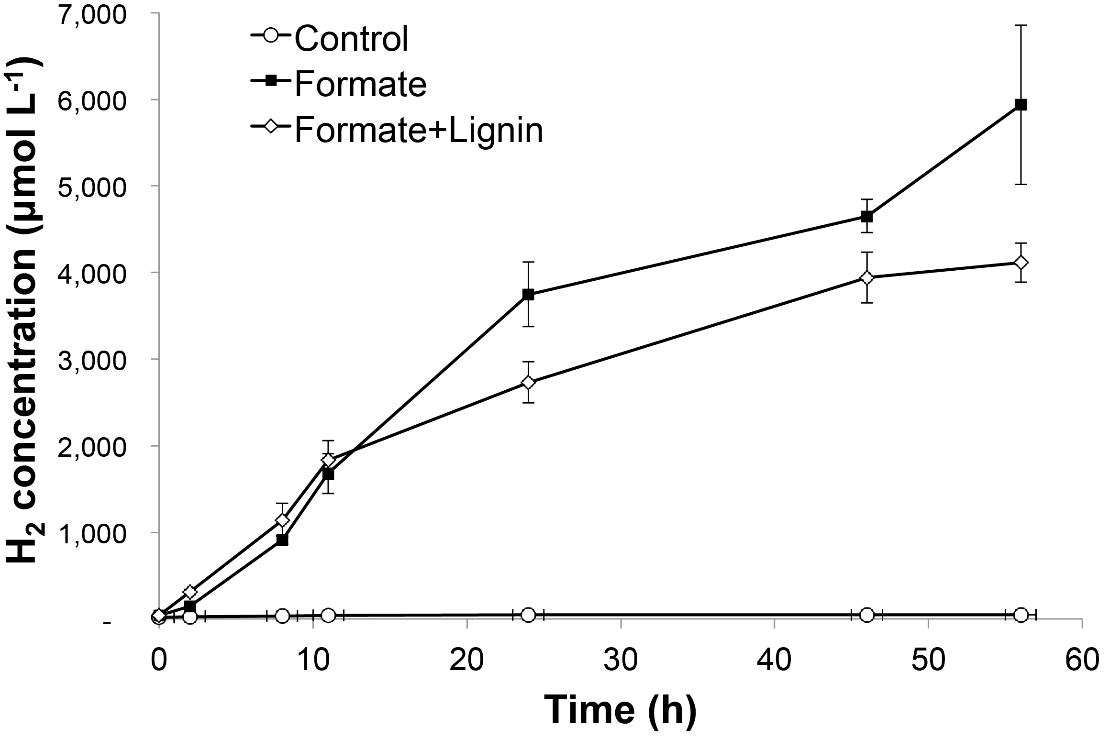
**

**S4 Figure**. Cell suspensions of SCF1 were introduced to minimal media amended with no carbon source (Control, open circles), 150 mM formate (Formate, closed squares), or 150 mM formate and 0.05 % lignin (Formate+Lignin, open diamonds). Cell suspensions were monitored over time for headspace accumulation of hydrogen gas (H_2_), which should have been produced as a result of formatehydrogenlyase activity.
