# Supplementary material for: Multi-time series RNA-seq analysis of Enterobacter lignolyticus SCF1 during growth in lignin-amended medium
Source: PLoS One. 2017 Oct 19;12(10):e0186440. doi: 10.1371/journal.pone.0186440 (PMC5648182; doi:10.1371/journal.pone.0186440)
Supplement: S1 Table — (DOCX) [file pone.0186440.s006.docx]

**S1 Table.** Summary of statistics of cell density as measured by optical density (OD600) and colony forming units (CFUs) for time points showing significant effect of lignin addition.

|  | OD600 | | CFUs | |
| --- | --- | --- | --- | --- |
| Time | Effect size^a^ | Percent change^b^ | Effect size | Percent change |
| 36h | 3.38 s.d. * | 5.1% | 2.02 s.d. * | 144% |
| 49h | 9.56 s.d. * | 20.5% | 0.104 s.d. | -4.59% |
| 60h | 10.2 s.d. * | 26.4% | 0.150 s.d. | -18.9% |

^a^ Effect size is calculated as Cohen’s d using pooled standard deviations. The calculated effect size is equivalent to standard deviation, and effect sizes greater than 2 (equivalent to a shift of two standard deviations) is considered significant in this analysis.

^b^ Percent change was calculated as the difference in cell biomass (by OD600 or CFUs) in lignin amended minus unamended conditions, divided by biomass in unamended conditions times 100%.
