# Supplementary material for: Multi-time series RNA-seq analysis of Enterobacter lignolyticus SCF1 during growth in lignin-amended medium
Source: PLoS One. 2017 Oct 19;12(10):e0186440. doi: 10.1371/journal.pone.0186440 (PMC5648182; doi:10.1371/journal.pone.0186440)
Supplement: S2 Table — Differential expression was defined as transcripts with adjusted p-values <0.05 and absolute value of log2 fold change >1 for these comparisons. (DOCX) [file pone.0186440.s007.docx]

**S2 Table.** Genes differentially expressed in early exponential (EE) phase during growth of SCF1 on lignin-amended versus unamended growth. Differential expression was defined as transcripts with adjusted p-values <0.05 and absolute value of log2 fold change >1 for these comparisons.

| Gene ID | Annotation | Gene name | Fold change in transcripts | | |
| --- | --- | --- | --- | --- | --- |
|  |  |  | EE | ME | ES |
| Large ribosomal subunits | | | | | |
| Entcl_0447 | Ribosomal protein L11 methyltransferase (EC 2.1.1.-) |  | 1.012 | 0.485 | 0.532 |
| Entcl_1971 | Ribosomal RNA large subunit methyltransferase A (EC 2.1.1.51) |  | 1.738 | -0.491 | 1.278 |
| Entcl_2707 | Ribosomal large subunit pseudouridine synthase C (EC 4.2.1.70) |  | 1.413 | -0.308 | -0.445 |
| Entcl_3015 | Ribosomal RNA large subunit methyltransferase F (EC 2.1.1.51) |  | 1.171 | 2.944 | 0.312 |
| Entcl_3667 | Ribosomal large subunit pseudouridine synthase A (EC 4.2.1.70) |  | -0.181 | 2.166 | 0.766 |
| Small ribosomal subunits | | | | | |
| Entcl_0386 | SSU ribosomal protein S12p (S23e) |  | 1.568 | -0.578 | 0.307 |
| Entcl_1957 | Ribosomal RNA small subunit methyltransferase F (EC 2.1.1.-) |  | 1.056 | -0.458 | 0.689 |
| Entcl_3759 | Ribosomal-protein-S18p-alanine acetyltransferase (EC 2.3.1.-) |  | 2.179 | 0.676 | 1.151 |
| Genes involved in cell biosynthesis | | | | | |
| Entcl_0457 | Rod shape-determining protein MreB | mreB | 1.423 | -1.616 | 0.102 |
| Entcl_0458 | Rod shape-determining protein MreC | mreC | 1.644 | -0.877 | 0.222 |
| Entcl_0459 | Rod shape-determining protein MreD | mreD | 1.497 | 0.0001 | -0.141 |
| Entcl_0460 | Septum formation protein Maf | Maf | 1.462 | 0.843 | 1.592 |
| Entcl_3162 | Rod shape-determining protein RodA | rodA | 1.062 | -0.476 | -0.332 |
| Entcl_0914 | Membrane-bound lytic mureintransglycosylase A precursor (EC 3.2.1.-) |  | 1.061 | 0.406 | 0.063 |
| Entcl_2735 | Lipid A biosynthesis lauroylacyltransferase (EC 2.3.1.-) |  | -0.388 | 1.392 | 0.086 |
| Entcl_4239 | Lipid flippase | wzxE | 1.054 | -0.378 | 0.237 |
| Entcl_2861 | Chromosome partition protein MukB | mukB | 0.329 | 1.245 | 1.426 |
| Entcl_2862 | Chromosome partition protein MukE | mukE | 0.278 | 3.199 | 0.778 |
| Entcl_2863 | Chromosome partition protein MukF | mukF | 0.570 | 2.329 | 0.363 |
| Entcl_1970 | Peptidoglycan synthetase (EC 2.4.1.129) |  | 0.281 | -0.290 | 1.412 |
| Entcl_3369 | Cell division trigger factor (EC 5.2.1.8) |  | 1.982 | -0.943 | 0.343 |
| Entcl_3370 | Cell division protein | bolA | -1.456 | 1.353 | 0.275 |
