# Supplementary material for: Multi-time series RNA-seq analysis of Enterobacter lignolyticus SCF1 during growth in lignin-amended medium
Source: PLoS One. 2017 Oct 19;12(10):e0186440. doi: 10.1371/journal.pone.0186440 (PMC5648182; doi:10.1371/journal.pone.0186440)
Supplement: S3 Table — Differential expression was defined as transcripts with adjusted p-values <0.05 and absolute value of log2 fold change >1 for these comparisons. (DOCX) [file pone.0186440.s008.docx]

**S3 Table**: Genes differentially regulated during growth related to the catabolism of sugars. Differential expression was defined as transcripts with adjusted p-values <0.05 and absolute value of log2 fold change >1 for these comparisons.

| Gene ID | Annotation | Gene name | Fold change in transcripts | | |
| --- | --- | --- | --- | --- | --- |
|  |  |  | EE | ME | ES |
| **Phosphoenolpyruvate phosphotransferase system (PTS)** | | | | | |
| Entcl_3690 | PTS system, mannose-specific IIB component (EC 2.7.1.69) |  | -1.162 | 4.245 | -0.216 |
| Entcl_3691 | PTS system, mannose-specific IIC component (EC 2.7.1.69) |  | -0.869 | 1.702 | 0.524 |
| Entcl_3692 | PTS system, fructose- and mannose-inducible IID component (EC 2.7.1.69) |  | -0.829 | 1.150 | 0.727 |
| Entcl_3811 | PTS system, mannose-specific IIA component (EC 2.7.1.69) |  | -2.264 | 2.128 | 0.120 |
| Entcl_3812 | PTS system, mannose-specific IIB component (EC 2.7.1.69) |  | -2.456 | 1.111 | 1.016 |
| Entcl_0175 | D-xylose transport ATP-binding protein XylG | xylG | -1.299 | 3.169 | -0.387 |
| Entcl_0176 | Xylose ABC transporter, periplasmic xylose-binding protein XylF | xylF | -1.121 | 2.840 | -0.898 |
| Entcl_0174 | Ribose ABC transport system, permease protein RbsC (TC 3.A.1.2.1) | rbsC | -1.406 | 2.224 | 0.170 |
| Entcl_1204 | Ribose/xylose/arabinose/galactoside ABC-type transport systems, permease component 1 |  | -1.370 | 2.661 | 0.654 |
| Entcl_1206 | Ribose/xylose/arabinose/galactoside ABC-type transport systems, ATP-binding protein (EC 3.6.3.17) |  | -1.337 | 3.212 | 0.795 |
| Entcl_4081 | Ribose ABC transport system, permease protein RbsC (TC 3.A.1.2.1) |  | -1.501 | 2.619 | 0.727 |
| Entcl_2546 | PTS system, cellobiose-specific IIA component (EC 2.7.1.69) |  | -2.466 | 0.508 | -1.015 |
| Entcl_3764 | PTS system, cellobiose-specific IIC component (EC 2.7.1.69) |  | -0.984 | 2.827 | 0.539 |
| Entcl_2547 | PTS system, chitobiose-specific IIC component (EC 2.7.1.69) |  | -1.155 | -0.518 | -1.129 |
| Entcl_2548 | PTS system, chitobiose-specific IIB component (EC 2.7.1.69) |  | -1.013 | 0.511 | -0.913 |
| Entcl_3261 | PTS system, maltose and glucose-specific IIC component (EC 2.7.1.69) |  | -1.106 | 0.023 | 0.342 |
| Entcl_4032 | PTS system, mannose-specific IIA component (EC 2.7.1.69) |  | -1.507 | -2.149 | -0.638 |
| Entcl_4033 | PTS system, mannose-specific IIB component (EC 2.7.1.69) |  | -0.983 | -1.816 | 1.150 |
| Entcl_4034 | PTS system, mannose-specific IIB component (EC 2.7.1.69) |  | -1.517 | -1.460 | -0.144 |
| Entcl_4036 | PTS system, mannose-specific IID component (EC 2.7.1.69) |  | -1.422 | -0.539 | 0.012 |
