# Supplementary material for: Multi-time series RNA-seq analysis of Enterobacter lignolyticus SCF1 during growth in lignin-amended medium
Source: PLoS One. 2017 Oct 19;12(10):e0186440. doi: 10.1371/journal.pone.0186440 (PMC5648182; doi:10.1371/journal.pone.0186440)
Supplement: S4 Table — Differential expression was defined as transcripts with adjusted p-values <0.05 and absolute value of log2 fold change >1 for these comparisons. (DOCX) [file pone.0186440.s009.docx]

**S4 Table.** Genes differential expressed during growth of SCF1 on lignin-amended versus unamended growth involved in the uptake of other sugars beside glucose. Differential expression was defined as transcripts with adjusted p-values <0.05 and absolute value of log2 fold change >1 for these comparisons.

| Gene ID | Annotation | Gene name | Fold change in transcripts | | |
| --- | --- | --- | --- | --- | --- |
|  |  |  | EE | ME | ES |
| Entcl_0166 | Ribose ABC transport system, permease (TC 3.A.1.2.1) | *rbsC* | -0.754 | 2.421 | -0.005 |
| Entcl_0167 | Ribose ABC transport system, ATP-binding protein (TC 3.A.1.2.1) | *rbsA* | 0.646 | 2.764 | 1.229 |
| Entcl_1205 | Ribose ABC transport system, permease protein (TC 3.A.1.2.1) | *rbsC* | -0.289 | 1.614 | -0.028 |
| Entcl_1207 | Ribose/xylose/arabinose/galactoside ABC-type transport systems, periplasmic sugar binding protein |  | 0.767 | 2.287 | 0.343 |
| Entcl_3382 | Ribose ABC transport system, ATP-binding protein RbsA (TC 3.A.1.2.1) |  | 0.460 | 1.739 | -0.273 |
| Entcl_3383 | Ribose ABC transport system, permease protein RbsC (TC 3.A.1.2.1) |  | 0.100 | 2.160 | -0.605 |
| Entcl_4082 | Ribose ABC transport system, periplasmic ribose-binding protein RbsB (TC 3.A.1.2.1) |  | 0.633 | -0.048 | 1.529 |
| Entcl_4174 | Ribose ABC transport system, ATP-binding protein RbsA (TC 3.A.1.2.1) |  | -0.821 | 1.065 | 0.113 |
| Entcl_4403 | Ribose ABC transport system, permease protein RbsC (TC 3.A.1.2.1) |  | 0.243 | 3.766 | 0.683 |
