# Supplementary material for: Multi-time series RNA-seq analysis of Enterobacter lignolyticus SCF1 during growth in lignin-amended medium
Source: PLoS One. 2017 Oct 19;12(10):e0186440. doi: 10.1371/journal.pone.0186440 (PMC5648182; doi:10.1371/journal.pone.0186440)
Supplement: S5 Table — Differential expression was defined as transcripts with adjusted p-values <0.05 and absolute value of log2 fold change >1 for these comparisons. (DOCX) [file pone.0186440.s010.docx]

**S5 Table**. Genes differentially expressed during growth of SCF1 on lignin-amended versus unamended growth. Differential expression was defined as transcripts with adjusted p-values <0.05 and absolute value of log2 fold change >1 for these comparisons.

| Gene ID | Annotation | Fold change in transcripts | | |
| --- | --- | --- | --- | --- |
|  |  | EE | ME | ES |
| Large ribosomal subunits | | | | |
| Entcl_0111 | LSU ribosomal protein L28p | 2.518 | -2.221 | -0.369 |
| Entcl_0112 | LSU ribosomal protein L33p , zinc-independent | 2.568 | -1.431 | 0.226 |
| Entcl_0394 | LSU ribosomal protein L3p (L3e) | 2.878 | -2.028 | -1.493 |
| Entcl_0395 | LSU ribosomal protein L4p (L1e) | 2.851 | -1.651 | -1.558 |
| Entcl_0396 | LSU ribosomal protein L23p (L23Ae) | 2.729 | -1.873 | -1.638 |
| Entcl_0397 | LSU ribosomal protein L2p (L8e) | 2.828 | -1.583 | -1.520 |
| Entcl_0399 | LSU ribosomal protein L22p (L17e) | 2.834 | -1.648 | -1.587 |
| Entcl_0401 | LSU ribosomal protein L16p (L10e) | 2.864 | -1.373 | -1.419 |
| Entcl_0402 | LSU ribosomal protein L29p (L35e) | 2.701 | -1.389 | -1.147 |
| Entcl_0404 | LSU ribosomal protein L14p (L23e) | 1.888 | -2.790 | -0.931 |
| Entcl_0405 | LSU ribosomal protein L24p (L26e) | 1.777 | -2.837 | -1.086 |
| Entcl_0406 | LSU ribosomal protein L5p (L11e) | 1.938 | -2.868 | -1.163 |
| Entcl_0409 | LSU ribosomal protein L6p (L9e) | 1.871 | -2.194 | -1.157 |
| Entcl_0410 | LSU ribosomal protein L18p (L5e) | 1.982 | -1.975 | -1.038 |
| Entcl_0412 | LSU ribosomal protein L30p (L7e) | 2.072 | -1.037 | -0.831 |
| Entcl_0413 | LSU ribosomal protein L15p (L27Ae) | 2.016 | -1.658 | -1.278 |
| Entcl_0415 | LSU ribosomal protein L36p | 1.153 | -0.735 | 0.635 |
| Entcl_0420 | LSU ribosomal protein L17p | 1.703 | -1.806 | -0.229 |
| Entcl_0479 | LSU ribosomal protein L13p (L13Ae) | 2.553 | -2.492 | -1.318 |
| Entcl_0511 | LSU ribosomal protein L27p | 2.103 | -3.920 | -0.769 |
| Entcl_1116 | LSU ribosomal protein L19p | 2.225 | -2.322 | -1.317 |
| Entcl_1518 | LSU ribosomal protein L25p | 1.733 | -2.556 | -0.631 |
| Entcl_2091 | LSU ribosomal protein L35p | 1.254 | -1.779 | -0.215 |
| Entcl_2092 | LSU ribosomal protein L20p | 1.360 | -1.101 | -0.306 |
| Entcl_2704 | LSU ribosomal protein L32p | 1.702 | -2.947 | -0.674 |
| Entcl_3961 | LSU ribosomal protein L9p | 2.224 | -2.216 | -1.153 |
| Entcl_4179 | LSU ribosomal protein L10p (P0) | 2.142 | -3.255 | -0.715 |
| Entcl_4180 | LSU ribosomal protein L1p (L10Ae) | 2.224 | -1.815 | -0.866 |
| Entcl_4181 | LSU ribosomal protein L11p (L12e) | 2.297 | -2.100 | -0.848 |
| Entcl_4338 | LSU ribosomal protein L31p, zinc-dependent | 2.562 | -2.655 | -0.601 |
| Entcl_4449 | LSU ribosomal protein L34p | 2.782 | -2.281 | -0.406 |
| Small ribosomal subunits | | | | |
| Entcl_0387 | SSU ribosomal protein S7p (S5e) | 1.669 | -1.200 | -0.096 |
| Entcl_0393 | SSU ribosomal protein S10p (S20e) | 2.837 | -2.065 | -1.523 |
| Entcl_0398 | SSU ribosomal protein S19p (S15e) | 2.262 | -1.743 | -1.574 |
| Entcl_0400 | SSU ribosomal protein S3p (S3e) | 2.811 | -1.708 | -1.132 |
| Entcl_0403 | SSU ribosomal protein S17p (S11e) | 2.272 | -2.350 | -0.411 |
| Entcl_0407 | SSU ribosomal protein S14p (S29e) | 1.933 | -2.103 | -0.677 |
| Entcl_0408 | SSU ribosomal protein S8p (S15Ae) | 1.829 | -2.196 | -1.298 |
| Entcl_0411 | SSU ribosomal protein S5p (S2e) | 2.011 | -1.743 | -0.918 |
| Entcl_0416 | SSU ribosomal protein S13p (S18e) | 1.760 | -1.522 | -0.235 |
| Entcl_0417 | SSU ribosomal protein S11p (S14e) | 1.568 | -2.446 | -0.852 |
| Entcl_0418 | SSU ribosomal protein S4p (S9e) | 1.635 | -2.051 | -0.321 |
| Entcl_0480 | SSU ribosomal protein S9p (S16e) | 2.373 | -2.242 | -0.951 |
| Entcl_0532 | SSU ribosomal protein S15p (S13e) | 2.009 | -1.511 | 0.435 |
| Entcl_0698 | SSU ribosomal protein S21p | 2.351 | -3.642 | -0.606 |
| Entcl_1113 | SSU ribosomal protein S16p | 2.214 | -2.881 | 1.415 |
| Entcl_2874 | SSU ribosomal protein S1p | 1.815 | -2.476 | -0.935 |
| Entcl_3568 | SSU ribosomal protein S2p (SAe) | 1.965 | -2.642 | -0.941 |
| Entcl_3761 | Ribosomal RNA small subunit methyltransferase C (EC 2.1.1.52) | 1.616 | -1.169 | 0.253 |
| Entcl_3962 | SSU ribosomal protein S18p, zinc-independent | 2.306 | -2.144 | -1.823 |
| Entcl_3964 | SSU ribosomal protein S6p | 2.202 | -2.448 | -1.594 |
