# Supplementary material for: Multi-time series RNA-seq analysis of Enterobacter lignolyticus SCF1 during growth in lignin-amended medium
Source: PLoS One. 2017 Oct 19;12(10):e0186440. doi: 10.1371/journal.pone.0186440 (PMC5648182; doi:10.1371/journal.pone.0186440)
Supplement: S6 Table — Differential expression was defined as transcripts with adjusted p-values <0.05 and absolute value of log2 fold change >1 for these comparisons. (DOCX) [file pone.0186440.s011.docx]

**S6 Table.** Genes differentially regulated during growth related to energy generation. Differential expression was defined as transcripts with adjusted p-values <0.05 and absolute value of log2 fold change >1 for these comparisons.

| Gene ID | Annotation | Gene name | Fold change in transcripts | | |
| --- | --- | --- | --- | --- | --- |
|  |  |  | EE | ME | ES |
| Entcl_1357 | Glucokinase (EC 2.7.1.2) |  | -0.219 | 1.745 | 0.905 |
| Entcl_3693 | Glucose-6-phosphate isomerase (EC 5.3.1.9) |  | -0.445 | 0.657 | 1.165 |
| Entcl_3694 | Glucose-6-phosphate isomerase (EC 5.3.1.9) |  | 0.334 | 0.404 | 2.008 |
| Entcl_2086 | 6-phosphofructokinase class II (EC 2.7.1.11) |  | -1.616 | 2.056 | -0.204 |
| Entcl_4346 | 6-phosphofructokinase (EC 2.7.1.11) |  | -0.908 | 1.207 | 1.267 |
| Entcl_0096 | Fructose-1,6-bisphosphatase, GlpX type (EC 3.1.3.11) |  | 1.174 | 1.245 | 1.911 |
| Entcl_4084 | fructose-bisphosphatealdolase, class II |  | -1.422 | 1.034 | -0.603 |
| Entcl_2022 | NAD-dependent glyceraldehyde-3-phosphate dehydrogenase (EC 1.2.1.12) |  | -1.471 | 1.979 | -0.441 |
| Entcl_0584 | 2-ketobutyrate formate-lyase (EC 2.3.1.-) / Pyruvate formate-lyase (EC 2.3.1.54) |  | -0.971 | 1.433 | -0.149 |
| Entcl_2993 | Pyruvate formate-lyase (EC 2.3.1.54) |  | -0.228 | 2.887 | 1.271 |
| Entcl_4043 | Pyruvate formate-lyase (EC 2.3.1.54) |  | 0.114 | 1.785 | -0.487 |
| Entcl_4296 | Pyruvate formate-lyase (EC 2.3.1.54) |  | 0.146 | 1.848 | 2.473 |
| Entcl_4297 | Pyruvate formate-lyase activating enzyme (EC 1.97.1.4) |  | -0.732 | 1.429 | 1.982 |
| Entcl_2992 | Pyruvate formate-lyase activating enzyme (EC 1.97.1.4) |  | -1.669 | 4.498 | 0.166 |
| Entcl_4042 | Pyruvate formate-lyase activating enzyme (EC 1.97.1.4) |  | -0.123 | 2.288 | -0.697 |
| Entcl_4288 | Pyruvate formate-lyase activating enzyme (EC 1.97.1.4) |  | -0.546 | 1.652 | 0.086 |
| Entcl_4087 | Formate dehydrogenase H (EC 1.2.1.2) |  | -0.600 | -5.075 | -2.652 |
| Entcl_2347 | Formate dehydrogenase N gamma subunit (EC 1.2.1.2) |  | -0.101 | 1.197 | 0.102 |
| Entcl_2348 | Formate dehydrogenase N beta subunit (EC 1.2.1.2) |  | 0.672 | 2.838 | 0.750 |
| Entcl_2349 | Formate dehydrogenase N alpha subunit (EC 1.2.1.2) (selenocysteine-containing) |  | -0.091 | 2.704 | 0.180 |
| Entcl_1432 | Phosphate acetyltransferase (EC 2.3.1.8) |  | 1.408 | -2.819 | -0.474 |
| Entcl_0583 | Propionate kinase (EC 2.7.2.15) / Acetate kinase (EC 2.7.2.1) |  | 0.227 | 3.281 | 0.840 |
| Entcl_1433 | Acetate kinase (EC 2.7.2.1) |  | 1.254 | -2.674 | -0.566 |
| Entcl_0976 | Formatehydrogenlyase transcriptional activator | fhlA | -0.423 | -3.441 | -2.039 |
| Entcl_0977 | [NiFe] hydrogenasemetallocenter assembly protein | hypE | -1.004 | -3.441 | -2.039 |
| Entcl_0978 | [NiFe] hydrogenasemetallocenter assembly protein | hypD | -0.958 | -4.863 | -0.775 |
| Entcl_0979 | [NiFe] hydrogenasemetallocenter assembly protein | hypC | -0.840 | -4.670 | -0.644 |
| Entcl_0980 | [NiFe] hydrogenase nickel incorporation-associated protein | hypB | -1.160 | -4.989 | -0.847 |
| Entcl_0981 | [NiFe] hydrogenase nickel incorporation protein | hypA | -0.952 | -7.137 | -2.261 |
| Entcl_0982 | Formatehydrogenlyase regulatory protein | hycA | -1.530 | -6.719 | -3.533 |
| Entcl_0983 | Formatehydrogenlyase subunit 2 | hycB | -1.631 | -6.407 | -2.854 |
| Entcl_0984 | Formatehydrogenlyase subunit 3 | hycC | -1.086 | -6.068 | -2.935 |
| Entcl_0985 | Formatehydrogenlyase subunit 4 | hycD | -0.783 | -5.963 | -3.890 |
| Entcl_0986 | Formatehydrogenlyase subunit 5 | hycE | -0.635 | -5.406 | -4.453 |
| Entcl_0987 | Ni,Fe-hydrogenase III medium subunit | hycF | -0.417 | -5.401 | -3.106 |
| Entcl_0988 | Formatehydrogenlyase subunit 7 | hycG | -0.441 | -4.890 | -4.195 |
| Entcl_0989 | Hydrogenase-4 component J | hyfG | -0.414 | -3.751 | -4.319 |
| Entcl_0990 | Coenzyme F420 hydrogenase maturation protease (EC 3.4.24.-) |  | -0.257 | -4.482 | -4.355 |
| Entcl_0994 | Fe-S-cluster-containing hydrogenase components 2 |  | -1.160 | -5.852 | -2.246 |
| Entcl_0995 | [NiFe] hydrogenasemetallocenter assembly protein HypF |  | -0.666 | -4.998 | -2.214 |
