# Supplementary material for: Multi-time series RNA-seq analysis of Enterobacter lignolyticus SCF1 during growth in lignin-amended medium
Source: PLoS One. 2017 Oct 19;12(10):e0186440. doi: 10.1371/journal.pone.0186440 (PMC5648182; doi:10.1371/journal.pone.0186440)
Supplement: S7 Table — Differential expression was defined as transcripts with adjusted p-values <0.05 and absolute value of log2 fold change >1 for these comparisons. (DOCX) [file pone.0186440.s012.docx]

**S7 Table**: Genes differentially regulated during growth related to other metabolic pathways. Differential expression was defined as transcripts with adjusted p-values <0.05 and absolute value of log2 fold change >1 for these comparisons.

| Gene ID | Annotation | Gene name | Fold change in transcripts | | |
| --- | --- | --- | --- | --- | --- |
|  |  |  | EE | ME | ES |
| Degradation of pectin | | | | | |
| Entcl_0602 | Hexuronate utilization operon transcriptional repressor | exuR | -0.496 | 3.216 | -0.035 |
| Entcl_0603 | Hexuronate transporter |  | -0.524 | 0.130 | 1.661 |
| Entcl_0604 | Uronateisomerase (EC 5.3.1.12) |  | -0.008 | 3.300 | -0.764 |
| Entcl_0605 | Altronate hydrolase (EC 4.2.1.7) |  | 0.916 | 3.537 | -0.301 |
| Entcl_0884 | 4-deoxy-L-threo-5-hexosulose-uronate ketol-isomerase (EC 5.3.1.17) |  | -0.716 | 1.855 | 0.762 |
| Entcl_0885 | 5-keto-D-gluconate 5-reductase (EC 1.1.1.69) |  | -0.028 | 2.055 | 1.277 |
| Entcl_1530 | D-mannonateoxidoreductase (EC 1.1.1.57) |  | 1.469 | 3.851 | 0.287 |
| Entcl_1531 | Mannonatedehydratase (EC 4.2.1.8) |  | -0.171 | 5.215 | 0.363 |
| Entcl_1892 | predicted 4-deoxy-L-threo-5-hexosulose-uronate ketol-isomerase (EC 5.3.1.17) |  | -0.610 | 2.070 | 0.544 |
| Entcl_2285 | Altronateoxidoreductase (EC 1.1.1.58) |  | 0.588 | 3.597 | -0.530 |
| Entcl_2997 | Hexuronate transporter |  | -0.629 | 3.447 | -0.077 |
| Entcl_3094 | Hexuronate transporter |  | -2.171 | 3.967 | 0.106 |
| Entcl_3467 | 6-phospho-3-hexuloisomerase |  | -1.251 | 0.763 | -0.776 |
| Chitin degradation | | | | | |
| Entcl_2924 | Chitinase (EC 3.2.1.14) |  | -1.358 | 1.622 | -0.565 |
| Entcl_2926 | Chitinase (EC 3.2.1.14) |  | -0.520 | 1.302 | -0.544 |
| Cellobiose degradation | | | | | |
| Entcl_0851 | 6-phospho-beta-glucosidase (EC 3.2.1.86) |  | -1.234 | 0.879 | 0.069 |
| Entcl_0991 | 6-phospho-beta-glucosidase (EC 3.2.1.86) | ascB | -0.915 | 1.496 | -0.362 |
| Entcl_1274 | 6-phospho-beta-glucosidase (EC 3.2.1.86) |  | 0.041 | 0.991 | -1.131 |
| Entcl_2544 | Chitobiose-specific 6-phospho-beta-glucosidase ChbF (EC 3.2.1.86) |  | -1.082 | 1.539 | -0.246 |
| Entcl_3004 | 6-phospho-beta-glucosidase (EC 3.2.1.86) |  | 0.437 | 2.680 | -0.150 |
