# Supplementary material for: Multi-time series RNA-seq analysis of Enterobacter lignolyticus SCF1 during growth in lignin-amended medium
Source: PLoS One. 2017 Oct 19;12(10):e0186440. doi: 10.1371/journal.pone.0186440 (PMC5648182; doi:10.1371/journal.pone.0186440)
Supplement: S8 Table — Differential expression was defined as transcripts with adjusted p-values <0.05 and absolute value of log2 fold change >1 for these comparisons. (DOCX) [file pone.0186440.s013.docx]

**S8 Table**: Genes differentially regulated along three measured growth phases. Differential expression was defined as transcripts with adjusted p-values <0.05 and absolute value of log2 fold change >1 for these comparisons.

| Gene ID | Annotation | EE | ME | ES |
| --- | --- | --- | --- | --- |
| Entcl_0025 | Multidrug resistance protein D | 2.409 | 1.076 | 3.520 |
| Entcl_0036 | Hexose phosphate uptake regulatory protein UhpC | 1.835 | 1.681 | 1.973 |
| Entcl_0043 | 2-aminoethylphosphonate:pyruvate aminotransferase (EC 2.6.1.37) | 3.202 | 3.934 | 1.390 |
| Entcl_0096 | Fructose-1,6-bisphosphatase, GlpX type (EC 3.1.3.11) | 1.174 | 1.245 | 1.912 |
| Entcl_0097 | ATP-dependent DNA helicase RecG (EC 3.6.1.-) | 1.295 | 3.181 | 1.935 |
| Entcl_0114 | Phosphopantetheine adenylyltransferase (EC 2.7.7.3) | 1.257 | 1.396 | 1.539 |
| Entcl_0143 | Mannitol operon repressor | 1.427 | 1.845 | 1.557 |
| Entcl_0165 | Ribose ABC transporter, periplasmic ribose-binding protein | 1.219 | 1.035 | 1.343 |
| Entcl_0192 | Outer membrane protein A precursor | 1.234 | 2.195 | 1.504 |
| Entcl_0198 | Phosphoethanolamine transferase specific for the outer Kdo residue of lipopolysaccharide | 1.785 | 2.335 | 1.542 |
| Entcl_0218 | GGDEF/EAL domain protein YhjH | 1.900 | 1.575 | 1.332 |
| Entcl_0317 | Transcriptional activator of maltose regulon, MalT | 1.264 | 1.112 | 1.246 |
| Entcl_0557 | Galactosamine-6-phosphate isomerase (EC 5.3.1.-) | 2.352 | 3.089 | 5.199 |
| Entcl_0614 | Autoinducer 2 (AI-2) ABC transport system, fused AI2 transporter subunits and ATP-binding component | 2.352 | 5.045 | 3.223 |
| Entcl_0623 | Evolved beta-D-galactosidase, beta subunit | 1.020 | 4.415 | 2.405 |
| Entcl_0629 | type 1 fimbriae major subunit FimA | 2.352 | 3.630 | 1.020 |
| Entcl_0736 | Probable Fe-S oxidoreductase family 2 | 1.517 | 1.037 | 1.107 |
| Entcl_1034 | Putative membrane protein | 1.066 | 1.089 | 1.137 |
| Entcl_1044 | Ribonucleotide reductase of class Ib (aerobic), beta subunit (EC 1.17.4.1) | 1.268 | 1.243 | 1.575 |
| Entcl_1160 | Putative outer membrane lipoprotein | 1.556 | 2.527 | 2.725 |
| Entcl_1172 | COG4123: Predicted O-methyltransferase | 1.849 | 2.765 | 2.586 |
| Entcl_1463 | O-succinylbenzoic acid--CoA ligase (EC 6.2.1.26) | 1.454 | 2.205 | 1.261 |
| Entcl_1483 | Ferredoxin | 2.204 | 2.519 | 1.560 |
| Entcl_2379 | Putative virulence factor | 1.308 | 2.866 | 1.146 |
| Entcl_2714 | Flagellar basal-body rod protein FlgG | 2.267 | 2.335 | 1.145 |
| Entcl_2973 | Multidrug translocase MdfA | 1.193 | 3.805 | 1.877 |
| Entcl_3051 | Adenosylmethionine-8-amino-7-oxononanoate aminotransferase (EC 2.6.1.62) | 1.442 | 2.805 | 2.055 |
| Entcl_3363 | Putative exported protein | 1.015 | 1.901 | 2.564 |
| Entcl_3597 | Putative PTS system IIA component yadI (EC 2.7.1.69) | 1.401 | 1.670 | 1.005 |
| Entcl_3622 | Type IV fimbrial assembly protein PilC | 1.321 | 4.100 | 1.282 |
| Entcl_3627 | Mutator mutT protein (7,8-dihydro-8-oxoguanine-triphosphatase) (EC 3.6.1.-) | 2.909 | 3.012 | 1.465 |
| Entcl_3858 | Periplasmic fimbrial chaperone StfD | 1.433 | 3.475 | 1.203 |
| Entcl_3880 | N-Acetyl-D-glucosamine ABC transport system, permease protein | 1.678 | 5.270 | 2.217 |
| Entcl_3881 | putative SN-glycerol-3-phosphate transport system permease | 1.227 | 3.322 | 1.198 |
| Entcl_3897 | Ornithine carbamoyltransferase (EC 2.1.3.3) | 1.359 | 2.401 | 5.952 |
| Entcl_3912 | PTS system, trehalose-specific IIB component (EC 2.7.1.69) / PTS system, trehalose-specific IIC component (EC 2.7.1.69) | 1.555 | 2.041 | 1.377 |
| Entcl_3917 | Ribonucleotide reductase of class III (anaerobic), large subunit (EC 1.17.4.2) | 2.832 | 1.176 | 1.708 |
| Entcl_3918 | Ribonucleotide reductase of class III (anaerobic), activating protein (EC 1.97.1.4) | 3.596 | 2.064 | 2.567 |
| Entcl_4068 | PhnG protein | 1.610 | 4.310 | 2.014 |
| Entcl_4069 | PhnH protein | 1.610 | 6.160 | 1.851 |
| Entcl_4121 | tRNA dihydrouridine synthase A (EC 1.-.-.-) | 2.018 | 1.292 | 2.067 |
| Entcl_4132 | Maltose/maltodextrin transport ATP-binding protein MalK (EC 3.6.3.19) | 3.941 | 3.777 | 2.288 |
| Entcl_4145 | Alpha-aspartyl dipeptidase Peptidase E (EC 3.4.13.21) | 1.240 | 3.202 | 1.219 |
| Entcl_4255 | Ketol-acid reductoisomerase (EC 1.1.1.86) | 3.395 | 2.283 | 2.258 |
| Entcl_4259 | Putative dihydroxyacetone kinase (EC 2.7.1.29), dihydroxyacetone binding subunit | 1.438 | 2.858 | 1.320 |
| Entcl_4269 | Acetolactate synthase large subunit (EC 2.2.1.6) | 2.153 | 1.230 | 2.266 |
| Entcl_4356 | L-rhamnose operon transcriptional activator RhaR | 1.373 | 3.979 | 1.294 |
| Entcl_4435 | Xanthine/uracil/thiamine/ascorbate permease family protein | 2.929 | 2.505 | 1.352 |
